# α-Synuclein-carrying astrocytic extracellular vesicles in Parkinson pathogenesis and diagnosis

**DOI:** 10.1186/s40035-023-00372-y

**Published:** 2023-08-25

**Authors:** Pan Wang, Guoyu Lan, Bin Xu, Zhenwei Yu, Chen Tian, Xia Lei, Wassilios G. Meissner, Tao Feng, Ying Yang, Jing Zhang

**Affiliations:** 1 Department of Pathology, The First Affiliated Hospital, Zhejiang University School of Medicine, Hangzhou, 310002 China; 2 National Human Brain Bank for Health and Disease, Zhejiang University, Hangzhou, 310002 China; 3 Department of Pathology, Peking University Health Science Center, Beijing, 100191 China; 4 Department of Neurology, Center for Movement Disorders, Beijing Tiantan Hospital, Capital Medical University, Beijing, 100070 China; 5 China National Clinical Research Center for Neurological Diseases, Beijing, 100070 China; 6 CNRS, IMN, UMR 5293, University of Bordeaux, 33000 Bordeaux, France; 7 CHU Bordeaux, Service de Neurologie des Maladies Neurodégénératives, IMNc, 33000 Bordeaux, France; 8 Department of Medicine, New Zealand Brain Research Institute, University of Otago, Christchurch, Christchurch, New Zealand

**Keywords:** Parkinson’s disease, Astrocyte, Extracellular vesicle, α-Synuclein, Lysosomal dysfunction

## Abstract

**Background:**

The accumulation of α-synuclein (α-syn), an essential step in PD development and progression, is observed not only in neurons but also in glia, including astrocytes. The mechanisms regulating astrocytic α-syn level and aggregation remain unclear. More recently, it has been demonstrated that a part of α-syn spreading occurs through extracellular vesicles (EVs), although it is unknown whether this process is involved in astrocytes of PD. It is known, however, that EVs derived from the central nervous system exist in the blood and are extensively explored as biomarkers for PD and other neurodegenerative disorders.

**Methods:**

Primary astrocytes were transfected with A53T α-syn plasmid or exposed to α-syn aggregates. The level of astrocyte-derived EVs (AEVs) was assessed by nanoparticle tracking analysis and immunofluorescence. The lysosomal function was evaluated by Cathepsin assays, immunofluorescence for levels of Lamp1 and Lamp2, and LysoTracker Red staining. The Apogee assays were optimized to measure the GLT-1^+^ AEVs in clinical cohorts of 106 PD, 47 multiple system atrophy (MSA), and 103 healthy control (HC) to test the potential of plasma AEVs as a biomarker to differentiate PD from other forms of parkinsonism.

**Results:**

The number of AEVs significantly increased in primary astrocytes with α-syn deposition. The mechanism of increased AEVs was partially attributed to lysosomal dysfunction. The number of α-syn-carrying AEVs was significantly higher in patients with PD than in HC and MSA. The integrative model combining AEVs with total and aggregated α-syn exhibited efficient diagnostic power in differentiating PD from HC with an AUC of 0.915, and from MSA with an AUC of 0.877.

**Conclusions:**

Pathological α-syn deposition could increase the astrocytic secretion of EVs, possibly through α-syn-induced lysosomal dysfunction. The α-syn-containing AEVs in the peripheral blood may be an effective biomarker for clinical diagnosis or differential diagnosis of PD.

**Supplementary Information:**

The online version contains supplementary material available at 10.1186/s40035-023-00372-y.

## Background

Parkinson’s disease (PD), with pathological hallmarks of the presence of aggregated α-synuclein (α-syn) in Lewy bodies (LB) and loss of dopaminergic neurons in the substantia nigra, is the second most common neurodegenerative disease after Alzheimer's disease (AD) and characterized by progressive motor and non-motor symptoms [1]. Clinically, it is challenging to distinguish PD from several diseases with overlapping symptoms, especially multiple system atrophy (MSA) [2, 3]. MSA is a rare neurodegenerative disease with pathological accumulation of α-syn inclusions in oligodendrocytes along with nigral neuronal loss, clinically characterized by a variable combination of progressive dysautonomia, cerebellar ataxia, and/or Parkinsonism [4–6]. The MSA-P type manifests with predominant parkinsonism, which is often mistaken for PD, especially at early disease stages [7]. Therefore, a crucial objective of the study was to find the molecular pathways or biomarkers separating the two conditions.

Although most investigations in PD and MSA are geared towards neurons and oligodendrocytes, respectively [8, 9], astrocytes, a main glial cell type in central nervous system (CNS), are also involved in the neurodegenerative processes of multiple diseases [10, 11]. For example, α-syn, the main culprit of the synucleinopathies, accumulates in astrocytes in PD with minimal reactive astrogliosis [12]. In contrast, reactive astrocytes in MSA remarkably increase without apparent α-syn aggregation in astrocytes [13]. Our previous report has indicated that abnormal astrocytic activation by pathological α-syn disrupts the blood–brain barrier (BBB) [14]. However, the source and clearance of glial pathological α-syn remain controversial. Clearly, astrocytes can phagocytose and degrade neuron-derived α-syn via the lysosomal pathway [15]. The accumulation of pathological α-syn in astrocytes disrupts normal cell homeostasis, including lysosomal and mitochondrial functions, as well as glutamate transport [16–18]. How glial cells might counteract this accumulation of intracellular α-syn remains largely unknown. Recently, it has been suggested that extracellular vesicles (EVs), including exosomes, are a crucial route for cells to dispose unwanted materials [19].

In addition to maintaining the homeostasis of multiple cellular systems, EVs are extensively investigated as a vehicle to carry biomarkers of various diseases [20, 21]. Indeed, we and others have reported that EVs derived from the CNS exist in peripheral blood and carry disease-related pathological proteins [22–24], serving as efficient biomarkers for neurodegenerative disorders. For example, we previously indicated that the level of α-syn in L1CAM-enriched neuron-derived EVs (NEVs) in the plasma of PD patients is higher than that of healthy controls (HCs) [25]. We also demonstrated that EVs secreted by oligodendrocytes are different between patients with PD and patients with MSA [26]. Moreover, we and others illustrated that blood EVs carrying synaptic function-related proteins contain different levels of AD pathological markers in AD patients compared with age-matched HCs [27, 28]. These studies suggest the possibility of identifying astrocyte-derived EVs (AEVs) in peripheral blood to assist the diagnosis of neurodegenerative diseases.

In this study, we began with the investigation of the effect and mechanism of α-syn on AEVs using primary astrocytes with overexpression of A53T α-syn or exposure to α-syn aggregates. Next, we demonstrated that AEVs in human blood did contain α-syn. To transform the detected α-syn-carrying AEVs into clinical applications, a sensitive and rapid flow cytometry-based technology (Apogee flow cytometry) was developed to measure the potential biomarkers distinguishing PD from MSA, as well as from age-matched neurologically HC individuals.

## Methods

### Human subjects and sample collection

Plasma samples from 256 subjects (103 age- and sex-matched neurologically HC individuals, 106 patients with PD and 47 patients with MSA) were obtained from Tiantan Hospital Affiliated to Capital Medical University for EV assessments. The diagnosis of ‘possible’ and ‘probable’ MSA was based on the second consensus criteria [6]. The ethics approval was obtained before study enrolment, and all participants signed written informed consent before blood sampling. Each blood sampling was conducted using the same procedure at the same condition. In particular, blood was collected into the BD Vacutainer® EDTA-coated tubes (367863, Franklin, USA) and centrifugated at 1500×*g* for 15 min to separate the plasma, followed by a second centrifugation at 3200×*g* for 15 min to remove cell debris. The plasma samples were aliquoted into 1.5-ml low protein binding tubes and stored at − 80 °C. A summary of the demographics and clinical data of the participants is provided in Table 1.

**Table 1:** Summary of the demographics and clinical data of participants

Group	HC	PD	MSA
*n*	103	106	47
Age^a^	56.5 ± 12.5	60.9 ± 13.9	61.2 ± 12.3
Sex, male: female	58:45	59:47	25:22
Disease duration, years^a^	N/A	6.4 ± 4.4^b^	3.2 ± 1.6^c^

*HC* healthy control, *PD* Parkinson’s disease, *MSA* multiple system atrophy

^a^Mean ± SD

^b^Sample of 93

^c^Sample of 36

### Astrocyte-derived EV isolation

EVs carrying astrocytic glutamate transporter 1 (GLT-1) were isolated as AEVs from human plasma following a previously established protocol [27]. In brief, 10 μg of anti-GLT-1 antibodies (MAB20001, Abnova) or normal Rabbit IgG (Santa Cruz Biotechnology, Dallas, TX) were incubated with Sulfo-NHS-LC-LC-biotin overnight at 4 °C. Twenty micrograms of biotin-conjugated antibodies were coated on one set (1 mg) of streptavidin-conjugated magnetic beads (Invitrogen, Waltham, USA) according to the manufacturer’s instructions. After quick thawing (within 2 min) at 37 °C, the plasma samples were centrifuged at 2000×*g* for 15 min and then at 12,000×*g* for 30 min at 4 °C. Then 500 μl supernatant was coated with 100 μl magnetic beads, diluted 1:1.5 with phosphate-buffered saline (PBS, pH 7.4), and incubated for 24 h with gentle rotation at 4 °C. The beads were then washed four times with 1 ml of 0.1% bovine serum albumin (BSA) in PBS and transferred to a new tube. Then 0.1 M glycine (pH 2.8) buffer was used to elute AEVs from the beads, which was further neutralized to pH 7.4 with a Tris buffer. For western blot, 40 μl loading buffer was added. For the Apogee detection of α-syn, 110 µl of 1.2% Triton-X-100  in PBS was added and shaken vigorously for 30 min. AEVs in clinical plasma samples were extracted in batches. Two identical reference plasma samples pooled from 30 HCs were added to each batch to assess batch variations. Comparable numbers of AEVs isolated from the reference plasma under different batches indicate an acceptable batch variation. The plasma samples with depletion of EVs (EV-poor plasma samples) were prepared by removing EVs after a two-step ultracentrifugation (100,000×*g* for 70 min at 4 °C, twice).

### Western blot analysis and immunoprecipitation

EV samples were prepared according to a previously published protocol [29]. The protein levels of the EVs were determined using a BCA assay kit (Thermo Scientific, Waltham, MA). The EV sample was mixed with the Laemmli sample buffer and denaturized at 95 °C for 5 min. Then 10 μg total protein of each sample was loaded and separated on an SDS-PAGE gel (Genescript, New Jersey, USA) before transferring to a PVDF membrane (Millipore, MA, USA). Then, the PVDF membrane was incubated with primary antibodies for 18 h at 4 °C, washed with TBST for 3 times (10 min each), and incubated with the HRP-conjugated secondary antibodies for 1 h at room temperature. After washing, the images were captured using a ChemiDoc XRS machine. The primary antibodies used in this study were anti-LC3I/II (14600-1-AP, Proteintech, Wuhan, China, 1:1000), anti-β-actin (20536-1-AP, Proteintech, 1:1000), anti-Alix (2171, Cell Signaling Technology, Danvers, MA, 1:1000), anti-CD9 (sc13118, Santa Cruz, 1:1000), anti-GFAP (3670s, Cell Signaling Technology, 1:1000), anti-GLT-1 (MAB20001, Abnova, 1:500), anti-Cathepsin L (CTSL) (55914, Cell Signaling Technology, 1:1000), anti-SQSTM1/p62 (ab109012, Abcam, Cambridge, UK, 1:1000), anti-pro-Caspase3 (A19654, ABclonal, Wuhan, China, 1:1000), and anti-GAPDH (G9545, Sigma Aldrich, St. Louis, MO, 1:5000).

For immunoprecipitation, EVs containing 500 μg total proteins were incubated with anti-GLT-1 (MAB20001, Abnova) overnight at 4 °C. Mouse brain tissue was used as the positive control. After addition of Protein A/G agarose (20 μl, sc-2003, Santa Cruz Biotechnology), the samples were subjected to rotation at 4 °C for 2 h. The PBS containing 0.05% Tween 20 (0.05% PBST) was used to wash the A/G agarose beads four times, and the proteins were eluted using 1 × Laemmli sample buffer for western blot analysis.

### Nanoparticle tracking analysis (NTA)

To optimize the number of particles counted for EVs derived from plasma and EVs immuno-enriched from primary astrocytes, the EVs were diluted with the PBS filtered through a 0.22-μm filter and analyzed using the NTA 3.2 software (Nanosight, Amesbury, UK). For each fraction, three repeated videos (60 s each) were captured, and all fractions were analyzed using the same threshold.

### Meso scale discovery (MSD) multiplexed immunoassays

The level of α-syn was detected by MSD following the procedures described previously [30]. Briefly, 0.05 μg of capture antibody (MJFR1, ab138501, Abcam) diluted in 50 μl PBS was added into the MSD plates and incubated for 1 h with 600 rpm shaking. After washing three times with 150 μl of 0.05% PBST, the plate was blocked with 100 μl D35 solution for 1 h with shaking followed by addition of 50 μl sample and calibrator (recombinant α-syn, Sino Biological, Beijing, China) per well, followed by a 2-h incubation. The plates were then washed again and incubated with the Sulfo-tag-labeled α-syn detection antibody (BD42, 610786, BD Biosciences, CA, USA) for 1 h with shaking. After washing, 150 μl of reading buffer (2×) was added to each well and the level of α-syn was detected by the MESO QuickPlex SQ 120 instrument.

### Apogee flow cytometry

The GLT-1 antibody was labeled with the Alexa Fluor 405 Rabbit IgG Labeling Kit, and the anti-α-syn (SYN211, ab206675, Abcam) and anti-α-syn aggregate (MJFR14, ab214033, Abcam) antibodies were labeled with the Alexa Fluor 488 Mouse/Rabbit IgG Labeling Kit, according to the manufacturer’s protocol. Twenty microliters of plasma were filtered using the  100-kDa filter, and then incubated with 200 μl of 0.1% Triton X-100 in PBS for 5 min. The plasma was then centrifuged at 12,000×*g* for 4 min, washed with 200 μl of PBS, and centrifuged again. The concentrated EVs were transferred to a new Eppendorf tube, added with  0.2-μg labeled GLT-1 antibody, and incubated for 30 min. Then, the labeled anti-α-syn (SYN211) and anti-α-syn aggregate (MJFR14) antibodies were added to the EVs and incubated for 30 min. After addition of  400-μl PBS, the labeled EVs were detected using Apogee flow cytometry.

### Preparation of α-syn aggregates

The aggregated α-syn was prepared following the protocol described previously [31]. Briefly, 1 μg/μl recombinant human α-syn protein (12093-HNAE, Sino Biological) was incubated with intermittent shaking cycles (5-min shaking at 200 rpm, 5-min resting) using a BioTek Synergy Neo2 (Agilent, California, USA) at 30 °C for 7 days. The α-syn aggregates were assessed through Dot blot, Thioflavin T (ThT) assay and transmission electron microscopy (TEM).

### Dot blot

Three microliters of the monomeric or aggregated α-syn sample with a total of 0.1 μg, 0.5 μg or 1 μg protein was loaded on the nitrocellulose membrane (Millipore). The membrane was then air-dried and incubated with an antibody specific for α-syn aggregates (MJFR14, ab214033, Abcam) for 2 h at room temperature after blocking with 1% BSA solution for 1 h. After washing with TBST 3 times, the HRP-conjugated secondary antibody was incubated for 1 h at room temperature. The images were taken using the LI-COR Odessey CLX machine.

### ThT assay

The ThT assay was used to detect the relative level of aggregated α-syn. Briefly, 1 μg/μl monomeric or aggregated α-syn and 10 μM ThT (596200, Sigma Aldrich) were dissolved in PBS (pH 7.4) with a total volume of 100 μl per well. The reaction was performed in a 96-well white plate (3610, Corning, New York, USA) and the relative ThT fluorescence signals were recorded using a BMG CLARIOstar Plus plate reader with λexc 450 nm and λem 480 nm.

### In vitro primary astrocyte and neuron cultures

The primary astrocytes were isolated from the cortex of a neonatal mouse. The meninges were removed, and the cortices were put into the cold DMEM. The tissues were then cut into small pieces and incubated with 0.25% EDTA-trypsin for 5 min at 37 °C. After centrifugation, the cells were filtered with a 70-μm cell mesh to remove the cell debris. The number of cells was counted and the cells were planted in a tissue flask  at a density of 1 × 10^6^/ml. When the astrocytes were confluent over 90%, the flasks were shaken at 220 rpm/min overnight at 37 °C, after which the primary astrocytes were collected.

The astrocytes raised in 2-ml DMEM with 10% fetal bovine serum (FBS) (density 1 × 10^6^/ml) were transfected with 1 μg of pcDNA3.1-A53T-SNCA using the lipofectamine™ 3000 transfection reagent (L3000015, Waltham, USA) or treated with 700 ng aggregated α-syn dissolved in  0.7-μl PBS for 24 h. An empty vector or PBS was used as a negative control. The astrocytes were then raised in FBS-free medium for another 24 h, and the medium was subsequently centrifuged at 2000×*g* for 15 min to remove cell debris. The AEVs in the supernatant of the medium were collected and examined using NTA.

Primary neurons were isolated from the neonatal mouse cortex. The neonatal mouse cortices were carefully dissected in ice-cold Hank’s balanced salt solution (Invitrogen). The tissue was then cut into small pieces and incubated with 0.25% EDTA-trypsin for 2 min at 37 °C. Next, the tissue was mechanically dissociated in DMEM using a sterile Pasteur pipette that had been fire-polished. The suspension was then centrifuged at 1000×*g* for 5 min at room temperature. Neuronal suspensions were plated at a density of 3 × 10^4^ cells/cm^2^ on poly-*D*-lysine-coated tissue culture dishes. Two days later, 2 μM of cytosine arabinoside was added to inhibit the proliferation of glial cells. The medium was half-replaced with fresh culture medium every three days. Experiments were conducted on the fifth day of culture.

### TEM

EVs were fixed with 4% PFA for 30 min, then 10-μl sample was dripped on the copper grids and incubated for 10 min, with 2 repeats for each sample. The excess liquid was carefully removed with filter paper. The sample was washed three times with PBS (pH 7.4) for 2 min each and then blocked with an appropriate blocking buffer (1% BSA and 5% normal goat serum [NGS]) for 30 min at room temperature. The anti-GLT-1 antibody was then added to the copper grids and incubated for 2 h. After washing, the colloidal gold-labeled secondary antibody was added to the copper grid and incubated for 1 h. The copper grids were then washed with PBS, fixed with 2.5% (*v*/*v*) glutaraldehyde for 10 min to stabilize the immunoreaction, washed 3 times with ddH_2_O, and then stained with 2% uranyl citrate for 2 min. The images were taken under a JEM-1400 PLUS microscope (JEOL, Tokyo, Japan). For the aggregated α-syn, 1 μg/μl monomeric or aggregated α-syn was mixed 1:1 (*v*/*v*) with 5% glutaraldehyde overnight at 4 °C for fixation. Then, the mixtures were layered onto copper grids and allowed to dry for 30 min. The samples were then stained with saturated uranyl citrate and 0.2% (*w*/*v*, pH 11) lead acetate for 30 min and washed three times with ddH_2_O before imaging on a HEM-1400 PLUS microscope (JEOL, Tokyo, Japan). Eight to ten TEM images were taken for each sample.

### Cathepsin activity assays

The relative activities of Cathepsin B (CTSB), Cathepsin D (CTSD), and CTSL were measured, respectively, using Cathepsin Activity Assay Kits (Fluorometric) (ab65300, ab65302, and ab65306; Abcam) following the manufacturer’s instructions. The CTSB assay kit employs a specific CTSB substrate sequence RR labeled with AFC. Consequently, the cleavage of the synthetic substrate RR-AFC and the subsequent release of free AFC can provide an indication for the CTSB activity. The CTSD assay kit utilizes a preferred CTSD substrate sequence GKPILFFRLK(Dnp)-D-R-NH2 labeled with MCA. The resulting fluorescence released from the substrate can reflect the level of CTSD activity. The CTSL assay kit utilizes a preferred CTSL substrate sequence FR labeled with AFC, with the released free AFC as an indicator of the CTSL activity. Briefly, 1 × 10^6^ astrocyte cells overexpressing pcDNA3.1-A53T-SNCA or exposed to aggregated α-syn were harvested and washed with 200-μl ice-cold PBS. The cells were resuspended in 200-μl chilled CD Cell Lysis Buffer on ice for 10 min and centrifuged at 12,000×*g* to remove large cell debris. Cell lysate (50 μl/well) was transferred into a 96-well plate and incubated with 50 μl of reaction buffer and 2 μl of substrate at 37 °C for 1 h. Samples were then quantified using a fluorescence plate reader (FlexStation 3, Molecular Devices, San Jose, CA) at Ex/Em = 328/460 nm.

### Immunofluorescence analysis

Astrocytes were fixed with 4% PFA for 15 min at room temperature, blocked with the blocking buffer (1% BSA, 0.3% Triton X-100, and 5% NGS in PBS) for 1 h at 4 °C, and incubated with primary antibodies diluted in the blocking buffer overnight at 4 °C. The primary antibodies used for immunofluorescence were anti-LC3 (14600-1-AP, Proteintech, Wuhan, China, 1:1000), anti-Lamp1 (24170, Abcam, 1:1000), anti-Lamp2 (199947, Abcam, 1:1000), anti-Alix (2171, Cell Signaling Technology, 1:1000), anti-GFAP (4674, Abcam, 1:1000), anti-α-syn (MJFR14, ab214033, Abcam, 1:100), and anti-α-syn (SYN211, ab206675, Abcam, 1:200). Secondary antibodies conjugated with Alexa Fluor 488, Alexa Fluor 555, or Alexa Fluor 647 (Abcam) were used at a dilution of 1:500. DAPI (Sigma Aldrich) was used for nuclear staining at 1:5000. Images were captured by an LSM FV3000 confocal microscope (Olympus, Tokyo, Japan) and quantified using the Image J software (Version 1.52a, NIH, Bethesda, MD).

### LysoTracker red staining

Astrocytes were cultured on glass coverslips and stained with 50 nM LysoTracker Red DND-99 (Thermo Fisher) for 30 min at 37 °C. Then, the cells were fixed with 4% PFA at room temperature, stained with DAPI, and examined under an LSM FV3000 confocal microscope (Olympus, Tokyo, Japan).

### Quantitative real-time PCR

Total RNA was extracted from primary cultured astrocytes or neurons using Trizol reagent (Invitrogen). Then 1 μg of RNA was transcribed into cDNA with the PrimerScript RT Reagent Kit (Thermo Scientific) following the manufacturer’s instructions. qPCR was performed using the PowerUp™ SYBR™ Green Master Mix (Applied Biosystems, Waltham, USA) in the CFX Connect Real-Time PCR Detection System (Bio-Rad). The expression level of *SNCA* was normalized to *GAPDH* and calculated based on the comparative cycle threshold Ct method (2^−ΔΔCt^). The following primers were used:

*SNCA* forward: 5’-CACTGGCTTTGTCAAGAAGGACC-3′, *SNCA* reverse: 5′-CATAAGCCTCACTGCCAGGATC-3′; *GAPDH* forward: 5′- CATCACTGCCACCCAGAAGACTG-3′, *GAPDH* reverse: 5′- ATGCCAGTGAGCTTCCCGTTCAG-3′.

### Statistical analysis

All analyses were performed in Prism 9.0 (GraphPad Software, La Jolla, CA) or SPSS 23.0 (IBM, Chicago, IL). Data were compared using two-tailed unpaired Student’s *t*-test or Mann–Whitney U-test (for two groups) or one-way ANOVA followed by a Tukey’s post-hoc test (for multiple groups). Receiver operating characteristic (ROC) curves for analytes were generated to evaluate their sensitivities and specificities in distinguishing PD from MSA or HC. The ‘optimal’ cut-off value for a ROC curve was defined as the value associated with the maximal sum of sensitivity and specificity.

## Results

### Increased release of astrocytic EVs induced by pathological α-syn

**Fig. 1:**
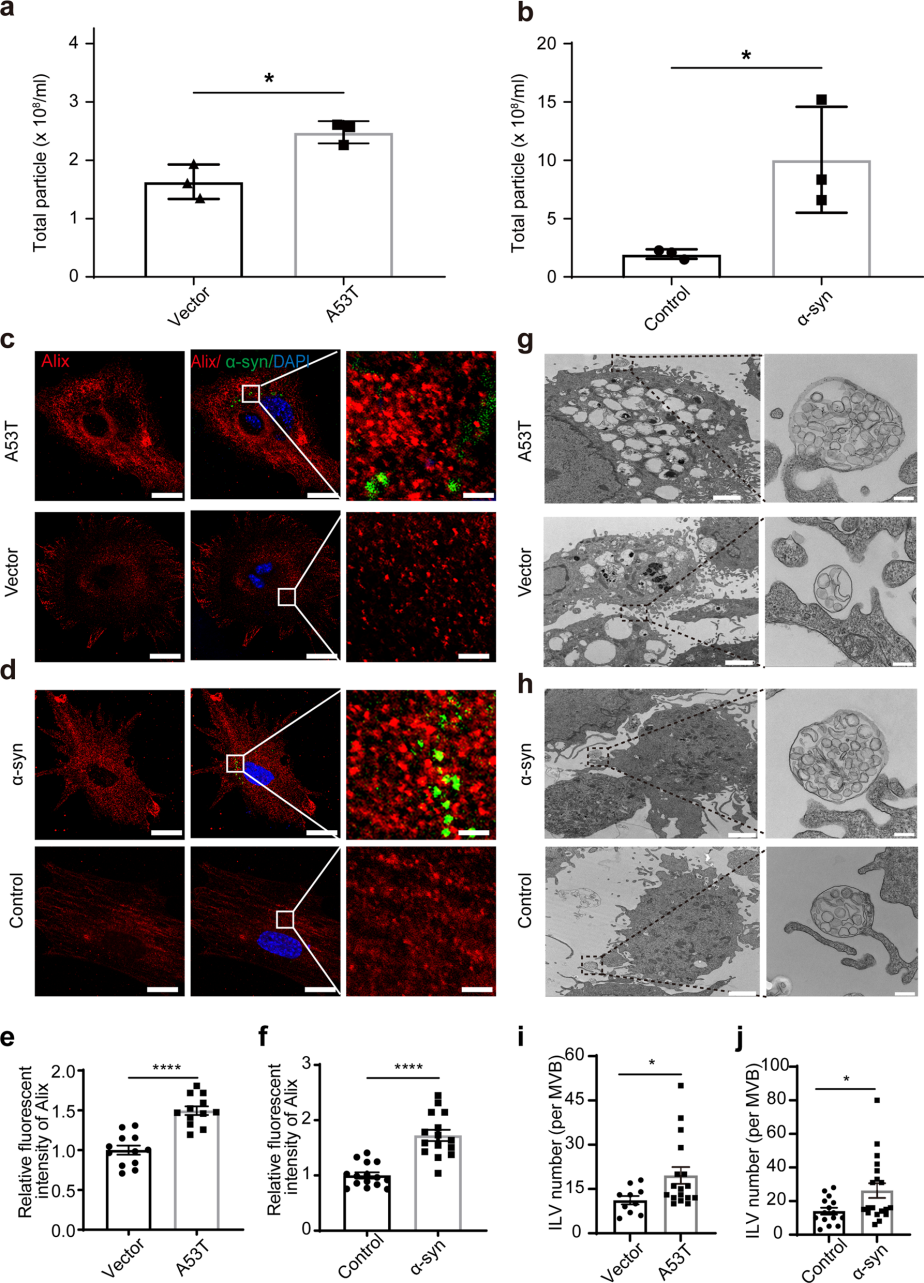
Increased release of astrocytic EVs induced by pathological α-syn in astrocytes. **a**, **b** The total number of EVs secreted from primary astrocytes overexpressing A53T α-syn or with exposure to aggregated α-syn for 24 h. *N* = 3 independent repeats. **c**, **d** Representative fluorescence images of primary astrocytes overexpressing A53T α-syn (**c**) or with exposure to aggregated α-syn (**d**) stained with Alix (red), α-syn (green) and nuclei (DAPI, blue). Scale bars, 20 μm for the left images and 2 μm for the right amplification images. **e**, **f** Quantification of the relative fluorescent intensity of Alix in primary astrocytes overexpressing A53T α-syn (**e**) or with exposure to aggregated α-syn (**f**). *N* = 10–15 astrocytes from 3 independent experimental repeats. **g**, **h** Representative TEM images of ILVs in MVBs of primary astrocytes overexpressing A53T α-syn (**g**) or with exposure to aggregated α-syn (**h**). Scale bars, 2 μm for the left images and 200 nm for the right amplification images. **i**, **j** Quantification of the number of ILVs in MVBs in primary astrocytes overexpressing A53T α-syn (**i**) or with exposure to aggregated α-syn (**j**). *N* = 10–15 astrocytes from 3 independent experimental repeats. Values are means ± S.E.M., unpaired *t*-test. *P < 0.05; ****P < 0.001

To investigate the pathological changes of astrocytic EVs in PD, primary astrocytes isolated from neonatal mouse cerebral cortex were transfected with A53T α-syn plasmid or exposed to α-syn aggregates to mimic the pathological state of PD (Additional file 1: Fig. S1a, b). The α-syn aggregates were generated and assessed with dot blot (Additional file 1: Fig. S1c), ThT assay (Additional file 1: Fig. S1d) and TEM (Additional file 1: Fig. S1e). NTA showed that the astrocytes overexpressing A53T α-syn or treated with α-syn aggregates secreted significantly more EVs compared to control (Fig. 1a, b), while the EVs secreted by astrocytes treated with α-syn insoluble fibrils were not significantly different from that of control (Additional file 1: Fig. S2a). In contrast to astrocytes, the exposure to α-syn aggregates had no influence on the neuronal secretion of EVs (Additional file 1: Fig. S2b). Meanwhile, the immunofluorescent results showed a significant increase in the level of Alix, a characteristic marker of EVs, in the astrocytes with overexpression of A53T α-syn or treatment with α-syn aggregates (Fig. 1c–f). Additionally, a type of EVs, multiple vesicular bodies (MVBs), which are single-membrane organelles containing intraluminal vesicles (ILVs) and essential for the biogenesis of exosomes [32], were also significantly elevated in the astrocytes with overexpression of A53T α-syn or exposure to α-syn aggregates (Fig. 1g–j). These results suggested that pathological α-syn aggregation could increase the EV secretion by astrocytes.

### Altered astrocytic EV secretion can be partially attributed to lysosomal dysfunction

**Fig. 2:**
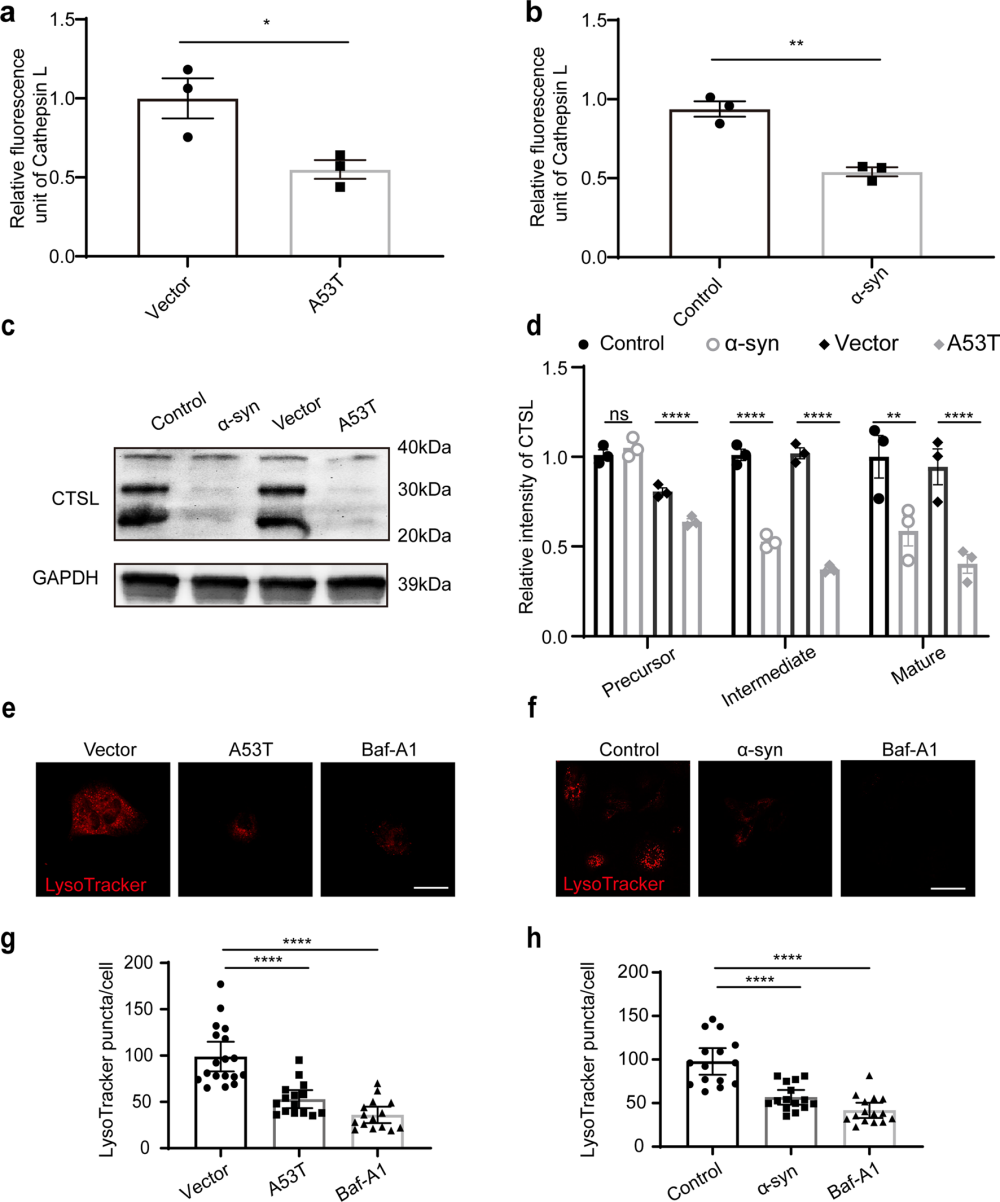
Altered astrocytic EV secretion can be partially attributed to lysosomal dysfunction. **a**, **b** Relative fluorescence unit of Cathepsin L (CTSL) in primary astrocytes overexpressing A53T α-syn or with exposure to α-syn aggregates. **c** The protein level of CTSL in astrocytes detected by western blotting. **d** Quantification of the protein levels of CTSL (including the precursor, intermediate, and mature forms) in astrocytes overexpressing A53T α-syn or with exposure to α-syn aggregates. *N* = 3 independent repeats. **e**, **f** Representative images of LysoTracker Red in astrocytes with overexpression of A53T α-syn (**e**) or α-syn aggregate exposure (**f**). **g**, **h** Quantification of the number of LysoTracker Red in astrocytes with overexpression of A53T α-syn (**g**) or α-syn aggregate exposure (**h**).* N* = 15–18 astrocytes from 3 independent experimental repeats. The astrocytes treated with 100 nM Baf-A1 were used as a positive control. Scale bars, 20 μm. Values are means ± S.E.M., unpaired *t*-test. *P < 0.05; **P < 0.01; ****P < 0.001

**Fig. 3:**
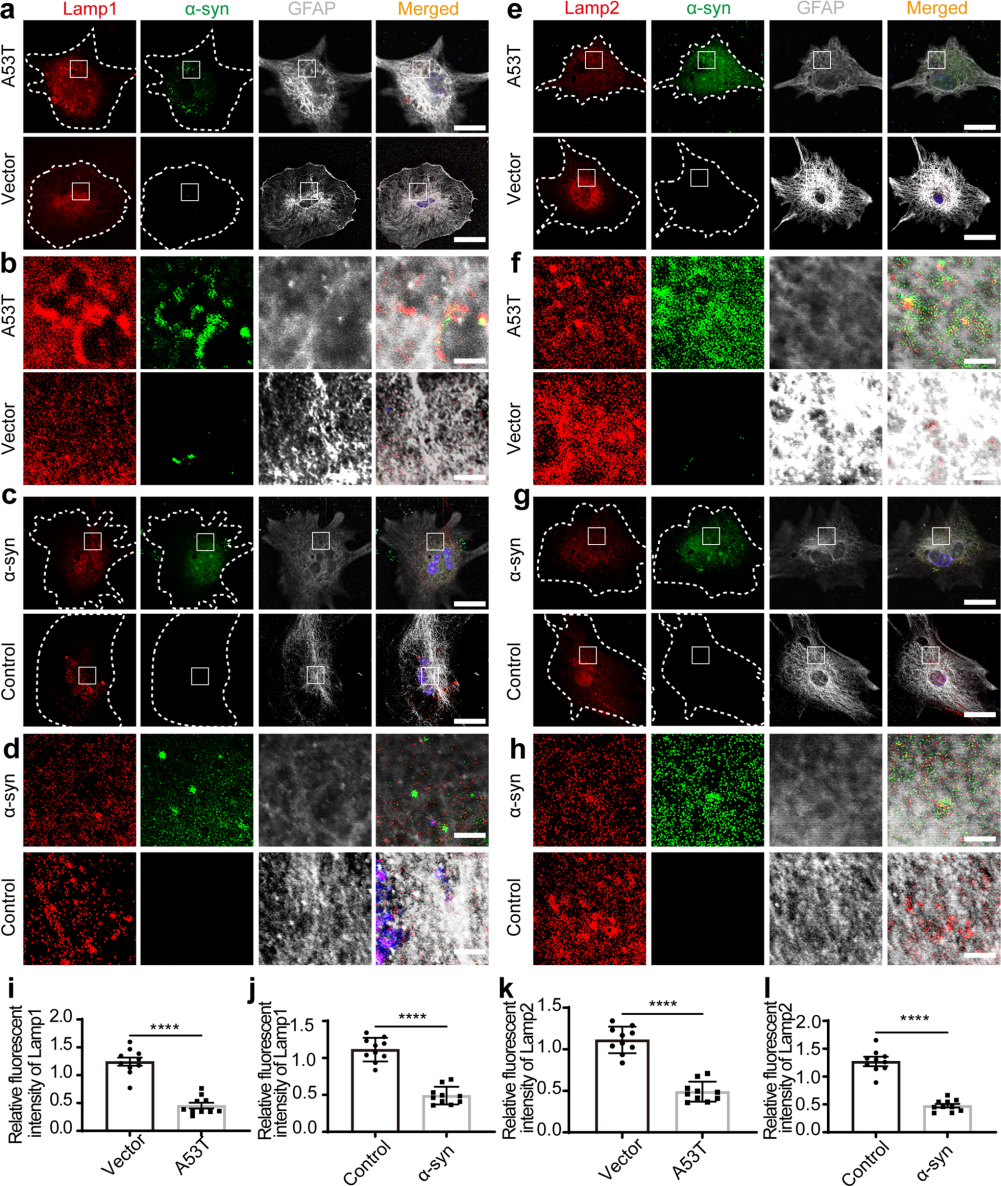
The levels of lysosome-associated membrane proteins decreased in astrocytes with pathological α-syn stimulation. **a**–**d** Representative fluorescence images of Lamp1 (red), α-syn (green), and nuclei (DAPI, blue) in astrocytes with overexpression of A53T α-syn or α-syn aggregate exposure. Scale bars, 20 μm. **e**–**h** Representative fluorescence images of Lamp2 (red), α-syn (green) and nuclei (DAPI, blue) in astrocytes with overexpression of A53T α-syn or α-syn aggregate exposure. Scale bars, 3 μm. **i**, **j** Quantification of the relative fluorescent intensity of Lamp1 in astrocytes with overexpression of A53T α-syn (**i**) or aggregated α-syn exposure (**j**). *N* = 10 astrocytes from 3 independent repeats. **k**, **l** Quantification of the relative fluorescent intensity of Lamp2 in astrocytes with overexpression of A53T α-syn (**k**) or aggregated α-syn exposure (**l**). *N* = 10 astrocytes from 3 independent repeats. Values are means ± S.E.M., unpaired *t*-test. *P < 0.05; **P < 0.01; ****P < 0.001

MVBs typically fuse with the plasma membrane to release exosomes or can be degraded through the lysosomal pathway [33]. With increased AEV release and MVB numbers, we hypothesized that the pathological α-syn accumulation in astrocytes may induce lysosomal dysfunction, therefore leading to increased secretion of AEVs. To assess lysosomal function, the activities of key lysosomal hydrolases, namely CTSB, CTSD, and CTSL, were evaluated. The activities of CTSB and CTSD were not influenced in astrocytes with overexpression of A53T α-syn or exposure to α-syn aggregates, compared with control (Additional file 1: Fig. S3a). However, the activity of CTSL in astrocytes with α-syn deposition was reduced significantly, indicating that α-syn may contribute to astrocytic lysosomal dysfunction (Fig. 2a, b). Western blot analysis showed that the protein levels of the precursor, intermediate, and mature forms of CTSL, all decreased significantly in primary astrocytes with overexpression of A53T α-syn or exposure to α-syn aggregates, compared with the vector or control group (Fig. 2c, d; Additional file 1: Fig. S4i, j). Consistent with these observations, the signal of LysoTracker Red dye, a probe sensitive to lysosomal pH, decreased significantly in astrocytes with overexpression of A53T α-syn or exposure to aggregated α-syn (Fig. 2e–h). Finally, the levels of Lamp1 and Lamp2, the major lysosome-associated membrane proteins [34], were examined in astrocytes by immunofluorescence. The results showed that the levels of Lamp1 and Lamp2 were significantly decreased in astrocytes with overexpression of A53T α-syn or exposure to α-syn aggregates when compared with the vector or control group (Fig. 3). Taken together, overexpression of A53T α-syn or exposure to α-syn aggregates decreased lysosomal function substantially, without inducing apparent astrocytic apoptosis (Additional file 1: Fig. S3b–d).

**Fig. 4:**
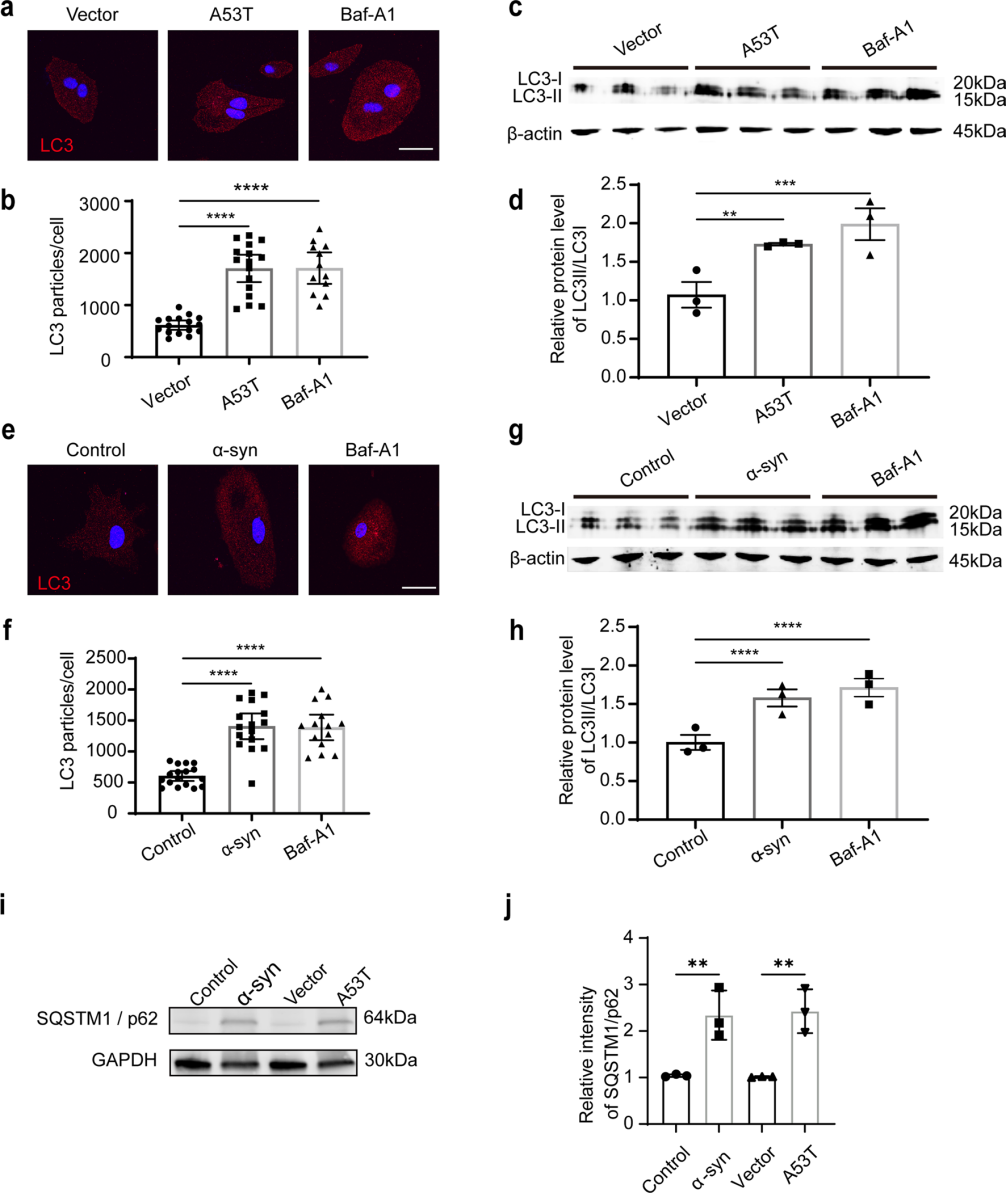
Increased autophagy in astrocytes with pathological α-syn stimulation. **a** Representative fluorescence images of LC3 (red) and nuclei (DAPI, blue) in astrocytes with overexpression of A53T α-syn. **b** Quantification of the LC3 particle number in astrocytes with overexpression of A53T α-syn. *N* = 12–16 astrocytes from 3 independent experimental repeats. **c**, **d** Western blots of LC3 in the astrocytes with overexpression of A53T α-syn and quantifications. *N* = 3 independent experimental repeats. **e** Representative fluorescence images of LC3 (red) and nuclei (DAPI, blue) in astrocytes treated with α-syn aggregates. **f** Quantification of the LC3 particle numbers in the astrocytes treated with α-syn aggregates. *N* = 12–16 astrocytes from 3 independent experimental repeats. **g**, **h** Western blots of LC3 in astrocytes treated with α-syn aggregates and quantifications. **i**, **j** Western blots of SQSTM1/p62 in the astrocytes with overexpression of A53T α-syn or treated with α-syn aggregates, and quantifications. *N* = 3 independent repeats. Values are means ± S.E.M., one-way ANOVA followed by a Tukey’s post-hoc test. *P < 0.05; **P < 0.01; ****P < 0.001

To further test the role of pathological α-syn in lysosomal dysfunction in astrocytes, the number of autophagosomes was assessed by the level of LC3-positive puncta, as cytosolic LC3 is relatively specifically associated with autophagosomes and autolysosomes [35]. The results showed that the level of LC3-positive puncta increased in astrocytes with overexpression of A53T α-syn or exposure to α-syn aggregates compared with the vector or control group (Fig. 4a, b, e, f). Western blot analysis further demonstrated that the protein levels of LC3I/II and SQSTM1/p62, another typical autophagy marker, increased in astrocytes with overexpression of A53T α-syn or exposure to α-syn aggregates compared with the vector or control group (Fig. 4c, d, g, h, i, j). As a positive control, Baf-A1 was performed to show that increased autophagy was associated with lysosomal dysfunction (Fig. 4). Taken together, these results further suggest that the increased secretion of AEVs induced by pathological α-syn in astrocytes can be partially attributed to lysosomal dysfunction.

### Detection of α-syn-carrying astrocytic EVs in human blood

**Fig. 5:**
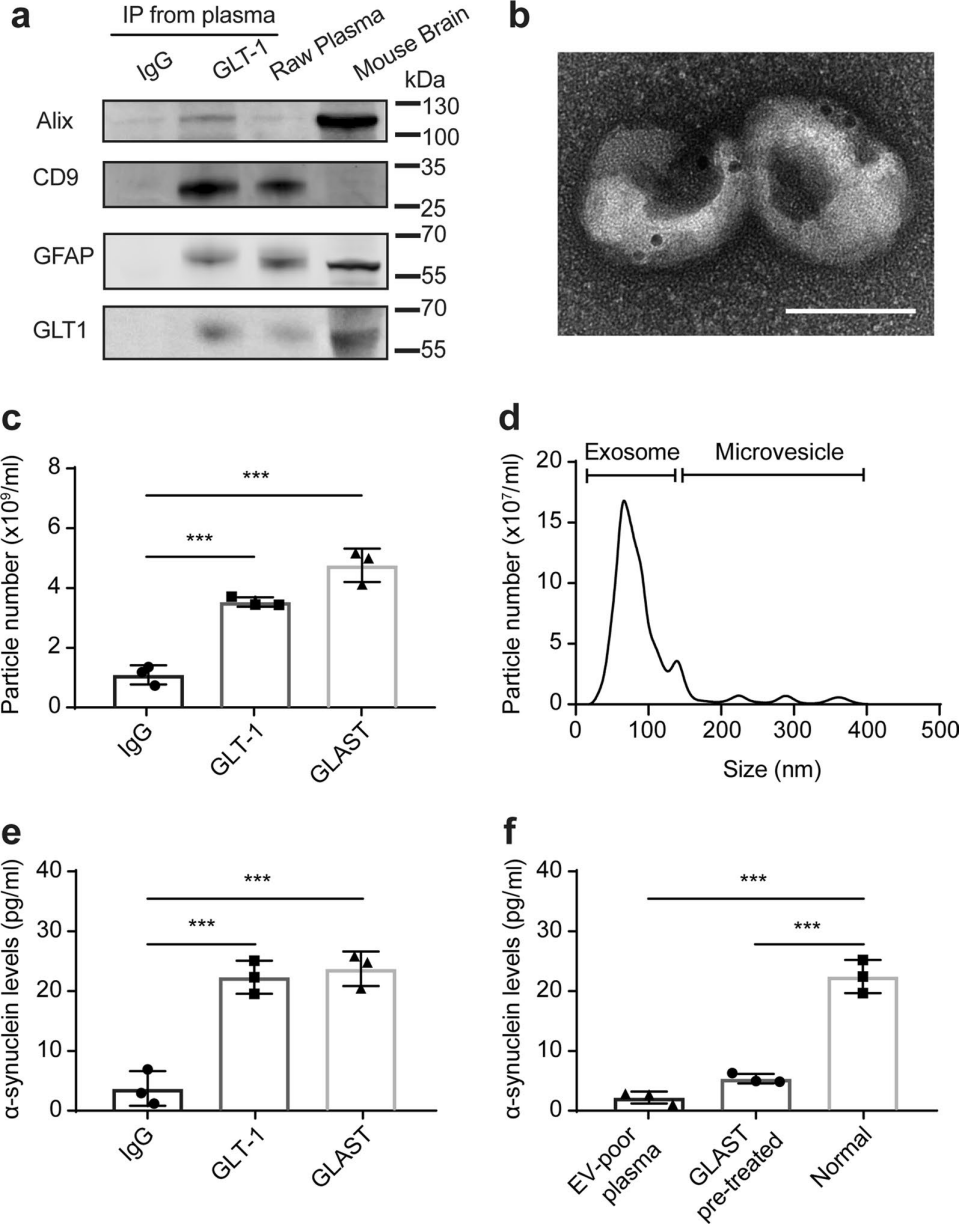
Characterization and quantification of GLT-1^+^ EVs in human plasma. **a** Representative western blot images showing the levels of Alix, CD9, GFAP and GLT-1 in the EVs immune-enriched by the normal mouse IgG or the anti-GLT-1 antibody from human plasma, in human raw plasma and mouse brain homogenates. **b** A representative TEM image showing GLT-1 protein on the surface of EVs immune-enriched by the anti-GLT-1 antibody from human plasma. Scale bar, 100 nm. **c** The number of EVs immune-enriched from human plasma by the anti-GLT-1 antibody, the GLAST antibody, or the normal mouse IgG antibody. *N* = 3 independent repeats. **d** The general size and distribution of isolated GLT-1^+^ EVs analyzed by NTA. **e** MSD analysis of the level of α-syn in the EVs immune-enriched from human plasma by the GLT-1 antibody, the GLAST antibody, or the normal mouse IgG antibody. *N* = 3 independent repeats. **f** MSD analysis of the level of α-syn in the EVs immune-enriched by the GLT-1 antibody from normal human plasma, EV-poor plasma, and plasma pre-captured by GLAST. *N* = 3 independent repeats. Values are means ± S.E.M., one-way ANOVA followed by a Tukey’s post-hoc test. ***P < 0.005

To probe the possibility of using the increased release of AEVs as a diagnostic marker of PD, we developed a strategy similar to that used to characterize neuron- and oligodendrocyte-derived EVs [25, 26], to define AEVs. To capture AEVs, GLT-1, a widely distributed excitatory amino acid transporter expressed almost exclusively in astrocytes in the CNS and responsible for the uptake of extracellular glutamate in brain tissue [36, 37], was selected based on our earlier preliminary observations (https://pss-system.cponline.cnipa.gov.cn/, WO2019153748A1). Western blot results showed that the GLT-1-captured EVs from plasma concurrently contained Alix and CD9, two typical exosome markers, as well as GFAP and GLT-1, two typical astrocytic markers, which were not detectable in the preparations enriched with the IgG isotype control (Fig. 5a). Under TEM, the GLT-1^+^ EVs showed a typical diameter of about 100 nm with immunogold labeling of GLT-1 protein on the membranes of EVs (Fig. 5b). The characteristics of GLT-1-enriched EVs were further quantified using NTA. The concentrations of GLT-1-enriched EVs were significantly higher than that of the IgG isotype control enriched preparations and comparable to that of the GLAST-enriched EVs, another common marker for AEVs [38] (Fig. 5c). Furthermore, NTA revealed that the sizes of GLT-1-enriched EVs mostly ranged from 30 to 150 nm, a well-known exosome range (Fig. 5d). These findings demonstrated that AEVs were present in the human peripheral blood and could be isolated by an anti-GLT-1 antibody. Considering the promoting role of α-syn in AEV secretion, another important question was whether these AEVs in plasma carried PD-related α-syn. For this purpose, the level of α-syn in AEVs was measured using a highly sensitive MSD immunoassay. The results showed that the AEVs enriched by GLT-1 or GLAST antibody indeed carried a significant amount of α-syn (Fig. 5e). To verify that the detected α-syn signal was indeed derived from the GLT-1-enriched AEVs rather than a contamination from the large amount of free α-syn in plasma, the EV-poor plasma with pre-depletion of EVs by ultracentrifugation, or the plasma pre-captured by GLAST, was prepared from the same batches of aliquots of the plasma. The results showed that the level of α-syn in GLT-1-enriched EVs from EV-poor plasma or GLAST-pre-captured plasma was significantly lower than that of normal plasma (Fig. 5f), further confirming the presence of α-syn in the GLT-1-enriched AEVs.

### Development of a flow cytometry-based assay for AEVs

**Fig. 6:**
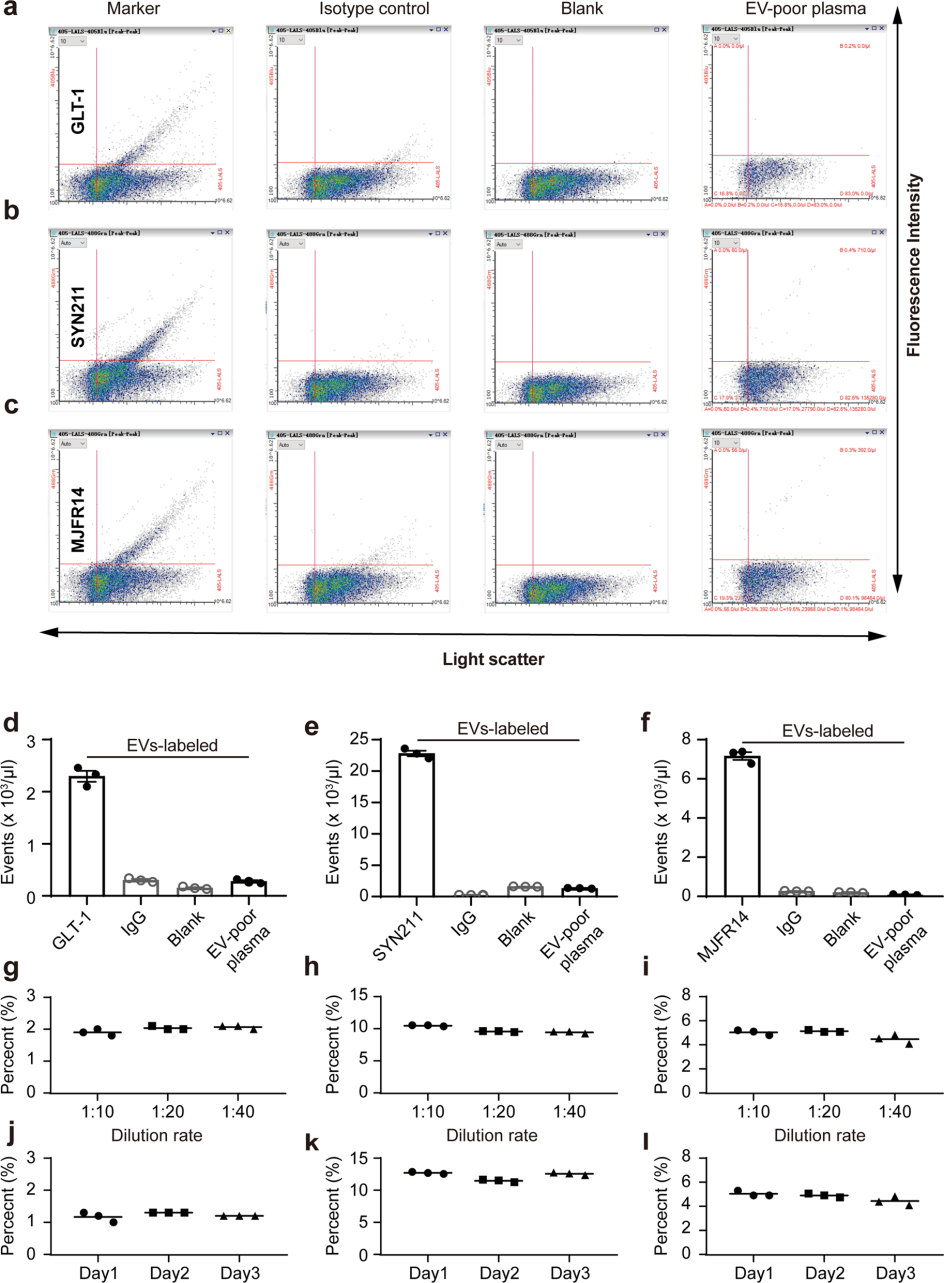
Development of a flow cytometry-based assay for astrocyte-derived EVs. **a–c** Representative histograms showing the populations of EVs positive for GLT-1, fluorophore-conjugated IgG isotype control, a blank (fluorophore only, no antibody) control experiment, and plasma with depletion of EVs positive for GLT-1 by ultracentrifugation. **b** Representative histograms showing the populations of EVs positive for SYN211, fluorophore-conjugated IgG isotype control, the blank control experiment, and plasma with depletion of EVs positive for SYN211 by ultracentrifugation. **c** Representative histograms showing the populations of EVs positive for MJFR14, fluorophore-conjugated IgG isotype control, the blank control experiment, and plasma with depletion of EVs positive for MJFR14 by ultracentrifugation. **d–f** Quantification data of positive EVs detected by the flow cytometry-based assay demonstrating the specificity of EV assays. **g**–**i** Linearity in different dilutions of EV plasma samples. **j**–**l** Stability of reference plasma (three replicates run each day on three separate days of the experiment for GLT-1, SYN211, and MJFR14)

To test the potential of plasma AEVs as a biomarker to differentiate PD from other forms of parkinsonism, the Apogee assays previously developed by us for CSF [39] and plasma [40] samples were optimized to measure the GLT-1^+^ and α-syn^+^ EVs in plasma. The results showed that GLT-1^+^ (coupled with Alexa Fluor 405) EVs were clearly detectable in plasma, with only a small amount of positive signal detected in the isotype IgG control group (Fig. 6a, d). Next, antibody for total α-syn (SYN211) or aggregate (MJFR14) was coupled with Alexa Fluor 488 to label different forms of α-syn on the EVs in plasma. The results demonstrated that the levels of EVs containing total α-syn (Fig. 6b, e) or aggregates (Fig. 6c, f) in plasma were significantly higher than those of the isotype IgG control. Meanwhile, flow cytometry analysis showed that the number of GLT-1^+^, SYN211^+^, or MJFR14^+^ particles in the EV-poor plasma was significantly reduced, supporting the specificities of the nanoscale flow cytometry assay (Fig. 6a–f). Finally, the assay stability and dilution linearity were also confirmed. The antibodies of GLT1, SYN211, and MJFR14 were diluted in three different ratios (1:10, 1:20, and 1:40) or incubated for three different incubation times (1 day, 2 days, and 3 days). The results showed that these antibodies had a stable labeling percentage of EVs with different dilution ratios (Fig. 6g–i) and different incubation times (Fig. 6j–l).

### Performance of AEV markers in the clinical cohort

**Fig. 7:**
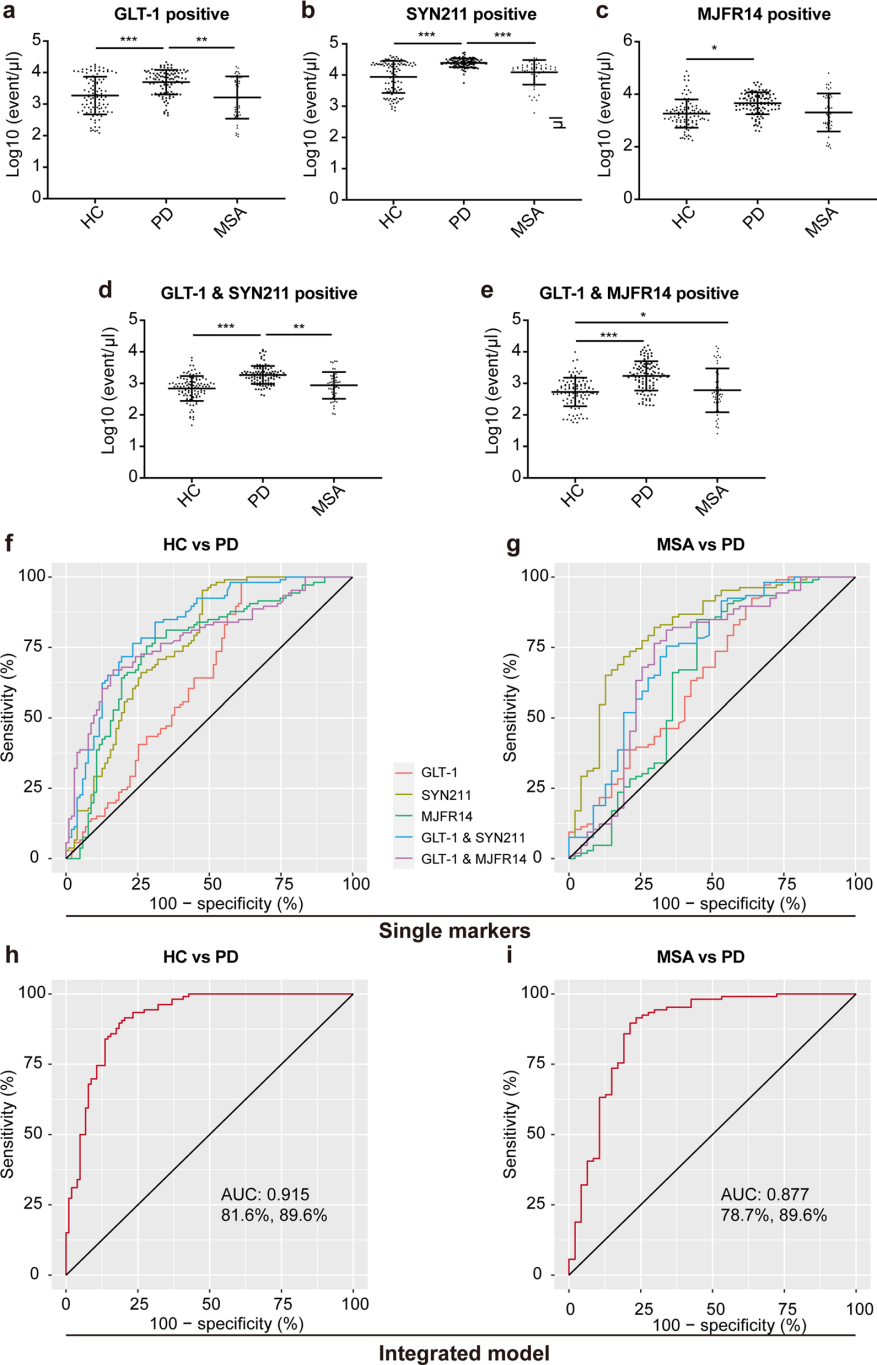
Flow cytometric analysis of astrocyte-derived EVs in the clinical cohort. **a** The number of GLT-1^+^ EVs was significantly higher in PD than in MSA or HC. **b** The number of SYN211^+^ EVs was significantly higher in PD than in MSA or HC. **c** The number of MJFR14^+^ EVs was significantly higher in PD than in HC. **d** The number of GLT-1^+^/SYN211^+^ EVs was significantly higher in PD than in MSA or HC. **e** The number of GLT-1^+^/ MJFR14^+^ EVs was significantly higher in PD and MSA than in HC. *N* = 32 in MSA group and 34 in PD group. **f** ROC curves showing the separation of PD from HC using EVs carrying GLT-1, α-syn, and α-syn aggregates. **g** ROC curves showing separation of PD from MSA using EVs carrying GLT-1, α-syn, and α-syn aggregates. **h** An integrative model including all EV markers distinguishes PD from HC. **i** An integrative model including all EV markers distinguishes PD from MSA. *N* = 103 in the HC group, 106 in the PD group, and 47 in the MSA group. Values are means ± S.E.M., one-way ANOVA followed by a Tukey’s post-hoc test. *P < 0.05; **P < 0.01; ***P < 0.005

With the optimized assays, clinical plasma samples from HC and patients with PD or MSA were examined blindly. Table 1 summarizes the characteristics of the clinical cohorts, with age and gender matched. As shown in Fig. 7a–c, the levels of GLT-1^+^, SYN211^+^, and MJFR14^+^ EVs in the PD group were all significantly higher than those in the HC group, while there was no significant difference between the MSA and HC groups. The levels of GLT-1^+^ and SYN211^+^ EVs in the PD group were remarkably higher than those in the MSA group, while the level of MJFR14^+^ EVs was not different between PD and MSA. Similarly, the level of AEVs carrying total α-syn (GLT-1^+^/SYN211^+^) in the PD group was dramatically higher than that in the HC or MSA group, while there was no significant difference between the HC and the MSA groups (Fig. 7d). The levels of AEVs carrying α-syn aggregates (GLT-1^+^/MJFR14^+^) in the PD and MSA groups were significantly higher than that in the HC group, while the PD group showed an increasing trend compared with the MSA group, yet with no statistical significance (Fig. 7e).

The ROC analysis was performed to evaluate the diagnostic performance of AEVs to differentiate patients with PD from HC or patients with MSA. Comparing PD and HC, the sensitivity and specificity were 76.4% and 77.4% (AUC = 0.8266, 95% CI 0.7708–0.8824) for GLT-1^+^/SYN211^+^ EVs and 67% and 84% (AUC = 0.785, 95% CI 0.7229–0.8470) for GLT-1^+^/MJFR14^+^ EVs (Fig. 7f), respectively. Comparing PD and MSA, the sensitivity and specificity were 75.5% and 66% (AUC = 0.7303, 95% CI 0.6360–0.8246) for GLT-1^+^/SYN211^+^ EVs and 81.1% and 66% (AUC = 0.7132, 95% CI 0.6122–0.8142) for GLT-1^+^/MJFR14^+^ EVs (Fig. 7g), respectively. The performance of GLT-1^+^, SYN211^+^, or MJFR14^+^ EVs alone was moderate in distinguishing PD from HC or MSA (Fig. 7f, g). Besides, the integrative model combining GLT-1^+^/SYN211^+^ EVs and GLT-1^+^/MJFR14^+^ EVs further increased the diagnostic power in differentiating PD from HC with an AUC of 0.915 (Fig. 7h, 95% CI 0.877–0.954, sensitivity = 81.6%, specificity = 89.6%) and from MSA with an AUC of 0.877 (Fig. 7i, 95% CI 0.807–0.946, sensitivity = 78.7%, specificity = 89.6%).

## Discussion

**Fig. 8:**
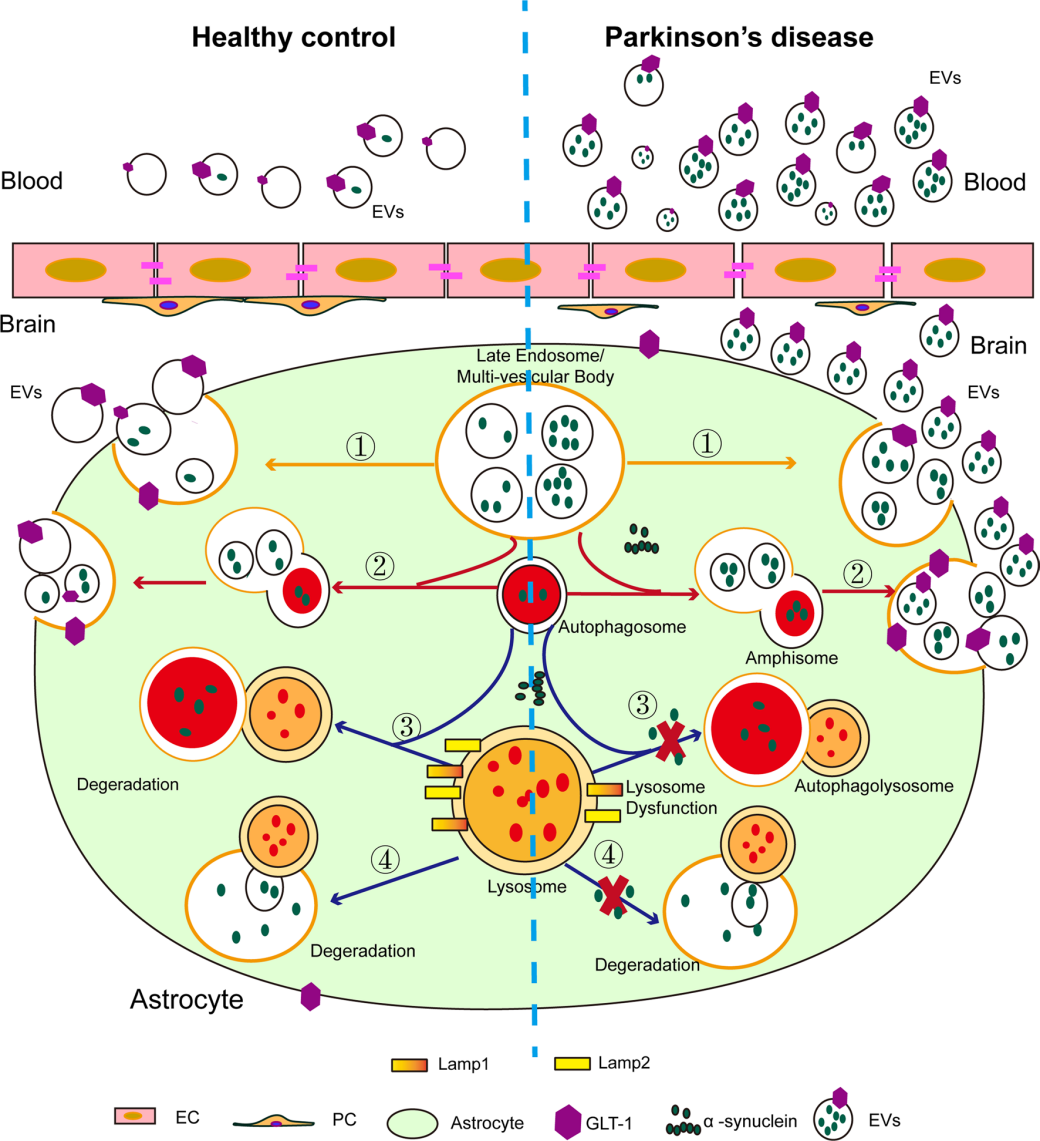
A schematic image showing α-syn-carrying astrocyte-derived EVs (AEVs) in Parkinson’s disease (PD) versus in healthy control. Pathological α-syn deposition could increase EV secretion by astrocytes, possibly through α-syn-induced lysosomal dysfunction. The α-syn-containing AEVs in peripheral blood may be an effective biomarker for clinical diagnosis or differential diagnosis of PD

The present study had several main findings regarding AEVs in PD (Fig. 8). First, astrocytic secretion of EVs increased with the pathological α-syn deposition in astrocytes. Second, the increased secretion of EVs was attributed, at least in part, to lysosomal dysfunction in astrocytes via processes involving dysfunction of autophagy and lysosome-associated membrane proteins. Finally, α-syn-carrying AEVs were not only detected in the peripheral blood plasma but also appeared to be effective in differentiating PD from MSA and HC.

Typically, astrocytes are thought to play a supportive role in neuronal metabolic homeostasis, e.g., providing nutrients and taking up secreted excitatory amino acids like glutamate, via the excitatory amino acid transporter GLAST/GLT-1 [41, 42]. In fact, astrocytes are critical for brain function as they actively interact with neurons, microglia, and other astrocytes, and are involved in BBB maintenance, neurotransmitter recycling, and energy homeostasis regulation [43]. Previous studies have reported that pathological α-syn accumulation appears substantially in the astrocytes of PD [44, 45], and internalization of α-syn in astrocytes may be involved in the progression of PD and associated neuroinflammation [46]. Increased astrocytic α-syn has been reported to be associated with several detrimental effects, including impairment of the BBB [47] as well as the accompanying neuronal damage [14]. In this study, we found that α-syn was expressed in astrocytes, though at a significantly lower level than that in neurons (Additional file 1: Fig. S2d) and pathological α-syn deposition in astrocytes could increase the EV secretion by primary astrocytes in the absence of obvious changes in lysosomal volume, cell viability, or the index of apoptosis. A previous study reported that α-syn-containing EVs secreted by astrocytes carrying the PD-related *LRRK2* G2019S mutation abnormally accumulated in neurites, which ultimately failed to support neuronal survival [48]. In addition, astrocytic EVs can be internalized by microglia, regulating the phagocytosis of microglia [49]. Besides the CNS, astrocytic EVs can cross the BBB and induce the transmigration of leukocytes to the brain in mice, thus mediating the communications of peripheral and CNS immune systems [50]. Thus, whether and how increased astrocytic secretion of EVs induced by pathological α-syn contributes to PD progression requires further investigation.

EVs can be generated via multiple mechanisms, and the biogenesis of exosomes can be initiated by the formation of MVBs [32]. MVBs typically fuse with the plasma membrane to release exosomes or alternatively fuse with lysosomes or autophagosomes to undergo degradation [33]. We revealed that with the astrocytic overexpression of A53T α-syn or exposure to aggregated α-syn, MVBs were larger and contained more ILVs and astrocytic release of EVs increased. Astrocytes with pathological α-syn stimulation exhibited reduced activity of CTSL, which partially reflects lysosomal activity. It should be noted that the extent of the observed decline in CTSL activity, measured in whole-cell lysate using the substrate preferred by CTSL, may differ from the reduction observed with a lysosomal-enriched fraction [51, 52]. Thus, we further investigated the lysosomal function of astrocytes upon pathological α-syn stimulation via evaluating the protein level of CTSL, performing LysoTracker staining, and examining the levels of lysosome-associated membrane proteins, namely Lamp1 and Lamp2. The combined findings support the notion that pathological α-syn may induce lysosomal deficits in astrocytes. In addition, the autophagosome-lysosome fusion measured by LC3 (an autophagosome marker) was accelerated in these cells, facilitating the accumulation of undigested autophagosomes. It should be noted that previous studies reported that lysosomal dysfunction could enhance EV secretion [53, 54]. Furthermore, it has also been reported that pathological α-syn aggregation is involved in autophagic-lysosomal pathway dysfunction [55, 56], which is intimately related to the metabolism of EVs. The secretion of aggregated pathological α-syn via EVs, the autophagy-lysosomal pathway, and the ubiquitin–proteasome pathway may be the complementary routes to remove the pathological α-syn from astrocytes.

BBB is known to limit the transport of nutrients and wastes selectively between the brain and blood; however, EVs derived from the CNS can cross the BBB readily and *vice versa* [57]. We previously demonstrated using Luminex assays that the neuron-derived exosomes or EVs could be transported from the brain to peripheral blood, and the level of α-syn contained in anti-L1CAM-captured EVs in plasma was significantly higher in patients with PD compared to HC [25]. A similar study in AD revealed that the CNS-derived EVs in plasma significantly decreased in AD compared to HC [27]. Here, we investigated whether AEVs can be detected in peripheral plasma and contain the traditional PD biomarker α-syn. For the isolation and identification of AEVs in plasma, a specific EV-surface marker for astrocytes is needed. Traditional astrocyte markers include GFAP, S100β, GLT-1, and GLAST. GLT-1 is highly expressed in the membrane of mature astrocytes [58]. Thus, GLT-1 was selected to isolate AEVs in plasma, and the specificity for AEV capture by GLT-1 antibody was verified by western blot, NTA, and TEM. Then, we evaluated the level of α-syn in GLT-1-positive AEVs using a highly sensitive MSD immunoassay, which revealed the presence of α-syn in GLT-1-enriched AEVs.

Next, we tested the potential of plasma AEVs as a biomarker to differentiate PD from MSA and HC in a clinical cohort using the Apogee assay. To avoid potential technical or subjective bias, the samples were analyzed by an investigator blind to the diagnosis of each sample, and the samples were batched based on diagnostic categories. The levels of AEVs carrying total α-syn and α-syn aggregate in the PD group were dramatically higher than those in the HC group. The level of AEVs carrying total α-syn in the PD group was noticeably higher than that in the MSA group. The sensitivities and specificities of plasma AEVs were 81.6% and 89.6% for differentiating PD patients from HC subjects, and 78.7% and 89.6% for differentiating PD patients from MSA patients. There was no correlation between the α-syn-carrying astrocytic EVs and gender when all PD patients were analyzed together (Additional file 1: Fig. S5a, b). One point worthy of noting is that as the number of patients with MDS-UPDRSIII score was limited in this study, the finding of no correlation between the level of AEVs carrying total α-syn (GLT-1^+^/SYN211^+^) or α-syn aggregate (GLT-1^+^/MJFR14^+^) and MDS-UPDRSIII score (Additional file 1: Fig. S5c, d) needs to be validated in the future. Another point relates to the fact that most of our patients recruited did not have a detailed record of total dopamine drugs received at the time of blood collection; therefore, a potential effect of variable amount of dopamine or related drugs on biomarker performance needs to be fully investigated in future studies.

Although larger cohorts and independent validations are needed to fully evaluate the biomarker potential of plasma AEVs and their protein cargos in future studies, quite a few implications emerged from this set of data. The most important implication is that α-syn-containing AEVs are able to distinguish PD patients from MSA patients. Future research is unquestionably required to study how differently AEVs are transported from the brain to the blood in PD versus MSA, in addition to establishing the molecular pathways driving astrocytic disease in PD. In PD, pathological α-syn mainly aggregates in neurons, while in MSA, pathological α-syn is mainly found in oligodendrocytes [7]. Given the pathological alterations in PD versus MSA, the levels of neuron-derived EVs were also investigated in this study. Of note, Apogee assay showed that the plasma level of neuron-derived L1CAM-positive EVs carrying total α-syn had no significant difference between PD and MSA (Additional file 1: Fig. S2c). Nonetheless, the possibility of neuron-derived EVs to distinguish PD patients from MSA patients needs to be investigated further.

In addition to the total and aggregated α-syn, biomarker studies on other α-synucleinopathy-related proteins in AEVs, including phosphorylated α-syn, DJ-1, and neurofilament light chain, which have shown biomarker potential in the CSF and blood [59, 60], may contribute to the diagnosis and differential diagnosis of PD. Moreover, α-syn has been reported to accumulate in subcortical astrocytes early in PD [13], and the distribution of α-syn-containing astrocytes is broader than LB [45]. Thus, considering the important role of astrocytes in PD, studies of AEVs in a prospective cohort in future experiments may improve the performance of the biomarker in early diagnosis of PD. Finally, astrocytes are activated at the site of Aβ deposition in AD [61, 62], and Aβ can be internalized by and detected in astrocytes in AD brains [63, 64]. This points to the potential of plasma AEVs as a biomarker to differentiate other neurodegenerative diseases.

## Conclusions

In summary, we demonstrated that the pathological α-syn aggregation could increase the astrocytic secretion of EVs. The increased AEV secretion is partially attributable to α-syn-induced lysosomal dysfunction. In addition, we isolated relatively astrocyte-specific or -enriched EVs in the plasma, and found that the levels of AEVs carrying total α-syn and α-syn aggregates are substantially higher in the PD group compared to HC. Furthermore, we revealed that the α-syn-containing AEVs may be a useful biomarker for the diagnosis and differential diagnosis of PD.

### Supplementary Information

**Additional file 1**. **Fig. S1**. Characterization of overexpression of A53T α-syn and aggregated α-syn in primary astrocytes. **Fig. S2**. The levels of neuron-derived L1CAM positive EVs carrying total α-syn in plasma of PD, MSA, and HC groups. **Fig. S3**. The effect of a-syn on the function of the lysosome in primary astrocytes. **Fig. S4**. Full-length western blots. **Fig. S5**. The correlation of the level of α-syn-carrying astrocytic EVs with gender as well as MDS-UPDRS III.

## Data Availability

All data generated or analyzed during this study are included in this published article and its supplementary information files.

## Abbreviations

α-Syn: α-Synuclein
CNS: Central nervous system
EV: Extracellular vesicle
AEV: Astrocyte-derived EV
LB: Lewy bodies
BBB: Blood-brain barrier
MVB: Multiple vesicular body
ILV: Intraluminal vesicle
GLT-1: Glutamate transporter-1
TEM: Transmission electron microscopy
ROC: Receiver operating curve
PD: Parkinson’s disease
AD: Alzheimer’s disease
MSA: Multiple system atrophy
HC: Healthy control
PBS: Phosphate-buffered saline
NTA: Nanoparticle tracking analysis
MSD: Meso scale discovery
ThT: Thioflavin T
BSA: Bovine serum albumin
NGS: Normal goat serum
CTSB: Cathepsin B
CTSD: Cathepsin D
CTSL: Cathepsin L
