# Supplementary material for: α-Synuclein-carrying astrocytic extracellular vesicles in Parkinson pathogenesis and diagnosis
Source: Transl Neurodegener. 2023 Aug 25;12:40. doi: 10.1186/s40035-023-00372-y (PMC10463943; doi:10.1186/s40035-023-00372-y)
Supplement: Supplementary file 1 — Additional file 1. Fig. S1. Characterization of overexpression of A53T α-syn and aggregated α-syn in primary astrocytes. Fig. S2. The levels of neuron-derived L1CAM positive EVs carrying total α-syn in plasma of PD, MSA, and HC groups. Fig. S3. The effect of a-syn on the function of the lysosome in primary astrocytes. Fig. S4. Full-length western blots. Fig. S5. The correlation of the level of α-syn-carrying astrocytic EVs with gender as well as MDS-UPDRS III. [file 40035_2023_372_MOESM1_ESM.docx]

**Additional file 1**

**
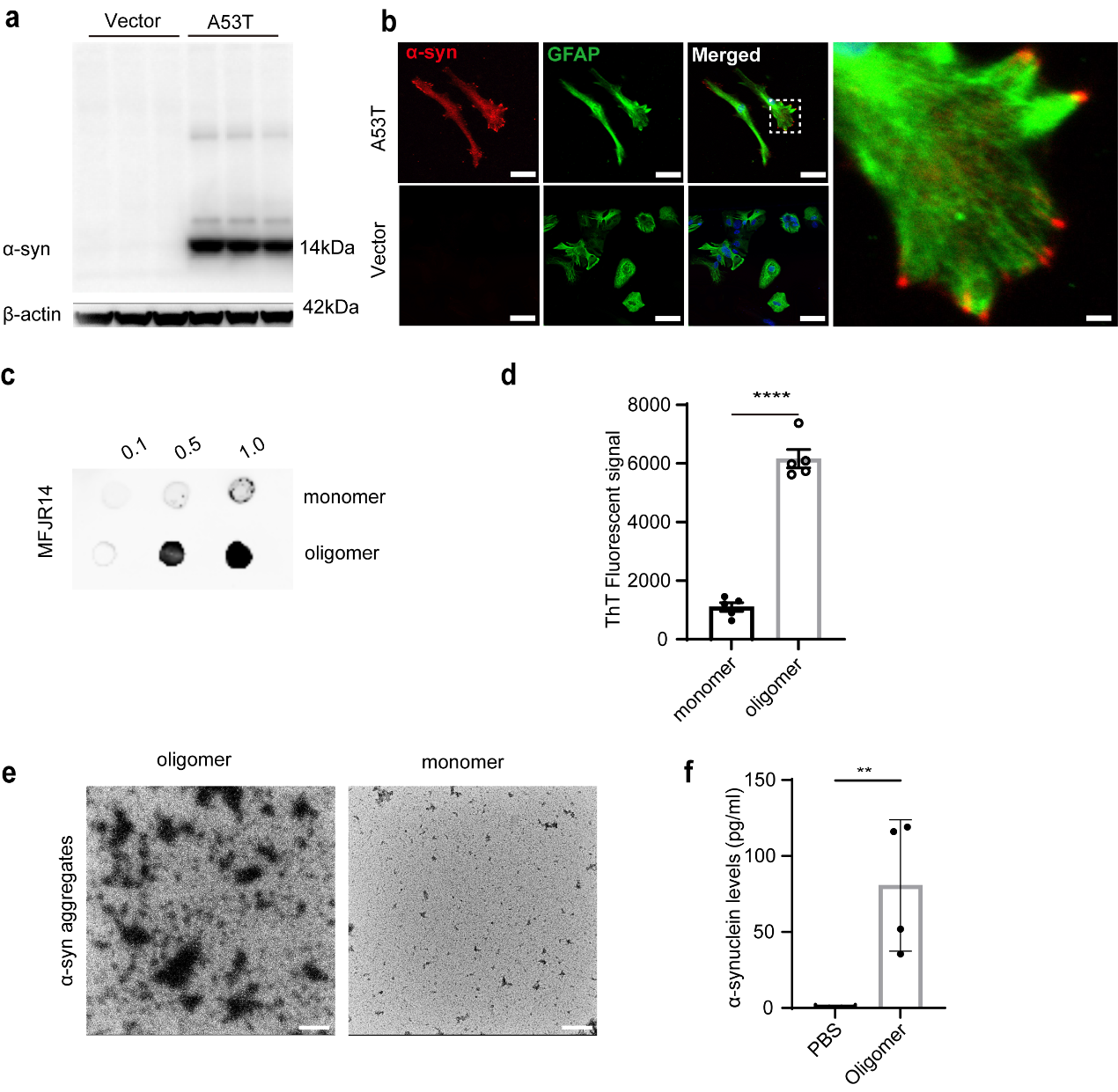
**

**Figure S1. Characterization of overexpression of A53T α-syn and aggregated α-syn in primary astrocytes.** (**a**) WB image showing the overexpressed A53T α-syn in primary astrocytes. (**b**) Representative immunofluorescence images showing the overexpressed A53T α-syn in primary astrocytes. Scale bar = 20 μm for left images and 2 μm for the right amplification image. (**c**) The dot blot image of aggregated α-syn stained with MJFR14 (Abcam, ab214033). (**d**) The relative THT fluorescence signals in monomeric and aggregated α-syn samples. (**e**) Representative TEM images of monomeric and aggregated α-syn. Scale bar = 200 nm. (**f**) The level of α-syn in primary astrocytes with exposure to α-syn and control vehicle detected by MSD. *N* = 4 independent repeats. Values are means ± S.E.M., unpaired t-test. ***P*<0.01; **** *P*<0.001.


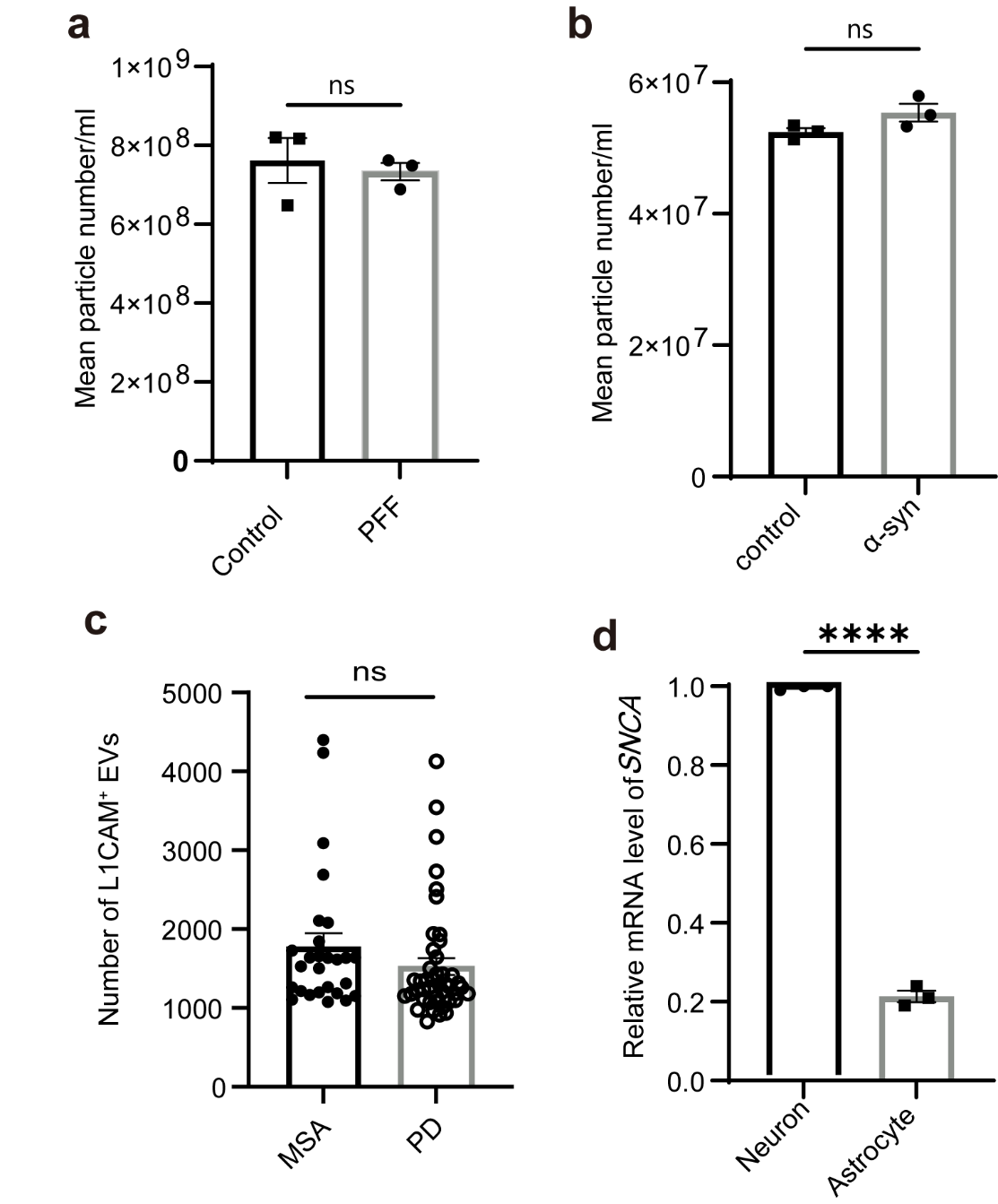


**Figure S2. The levels of neuron-derived L1CAM positive EVs carrying total α-syn in plasma of PD, MSA and HC groups. (a)** The level of EVs secreted by astrocytes treated with α-syn fibrils was not significantly different from that of control. *N =* 3 independent repeats. **(b)** The number of EVs released by primary neurons didn’t increase after aggregated α-syn exposure, compared with vehicle control. *N = 3* independent repeats. **(c)** The levels of neuron-derived L1CAM positive EVs carrying total α-syn in the PD group was not significantly different from those in MSA group. *N=32* in MSA group and 34 in PD group. **(d)** The relative mRNA of endogenous α-syn in primary astrocytes and neurons. *N = 3* independent repeats. Values are means ± S.E.M., unpaired t-test. ns, not significant; *****P*<0.001.


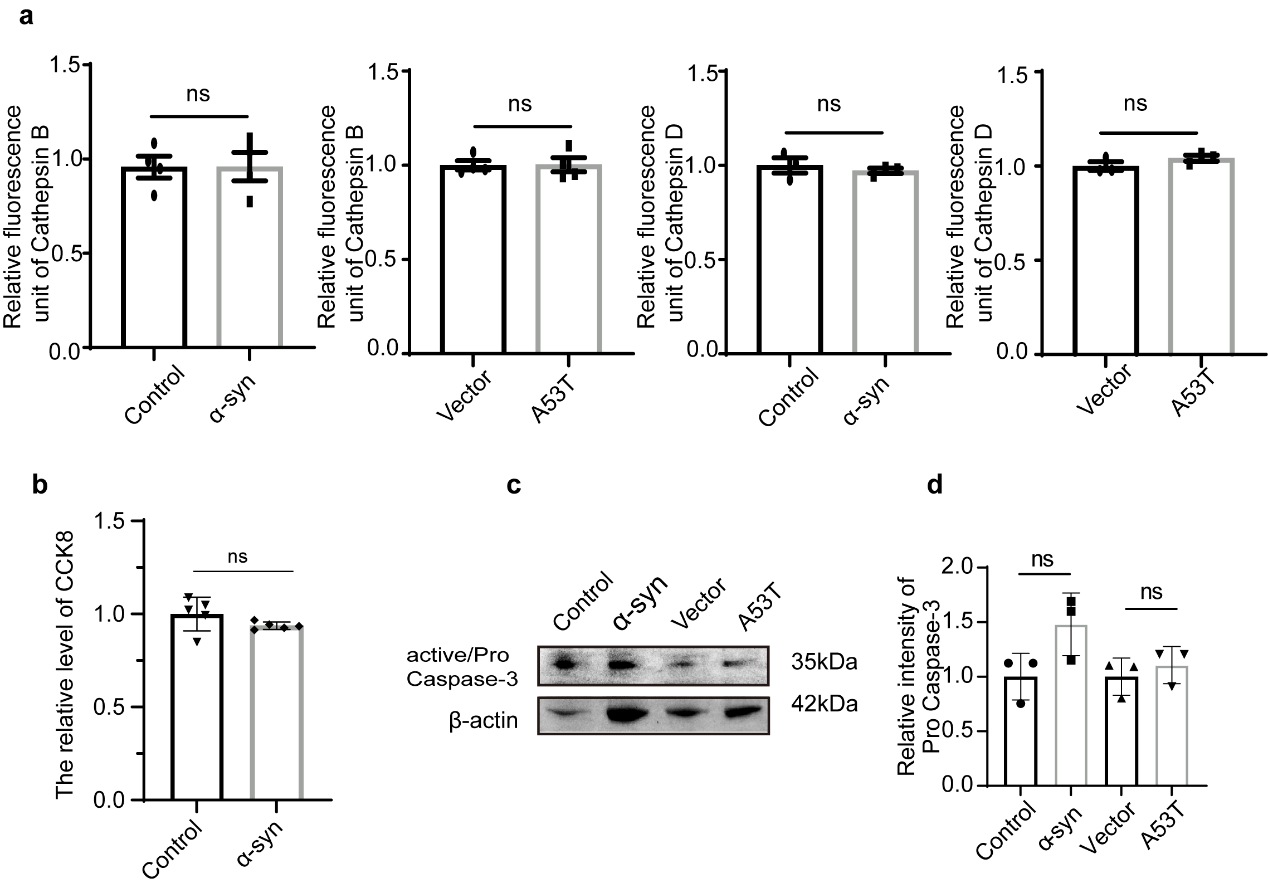


**Figure S3. The effect of a-syn on the function of the lysosome in primary astrocytes. (a)** Quantification of the relative fluorescence unit of Cathepsin B and Cathepsin D in astrocytes with overexpression of A53T α-syn or α-syn aggregates exposure. *N* = 4 independent repeats. (**b**) The relative level of CCK8 in astrocytes exposed to aggregated α-syn. *N* = 5 independent repeats. Values are means ± S.E.M., unpaired t-test. ns, not significant; ***P*<0.01; *****P*<0.001. (**c**) The protein level of active/Pro Caspase-3 in the astrocytes with overexpression of A53T α-syn or α-syn aggregates exposure detected by WB. **(d)** Quantification of the protein levels of active/Pro Caspase-3 in astrocytes with overexpression of A53T α-syn or α-syn aggregates exposure. *N* = 3 independent repeats.


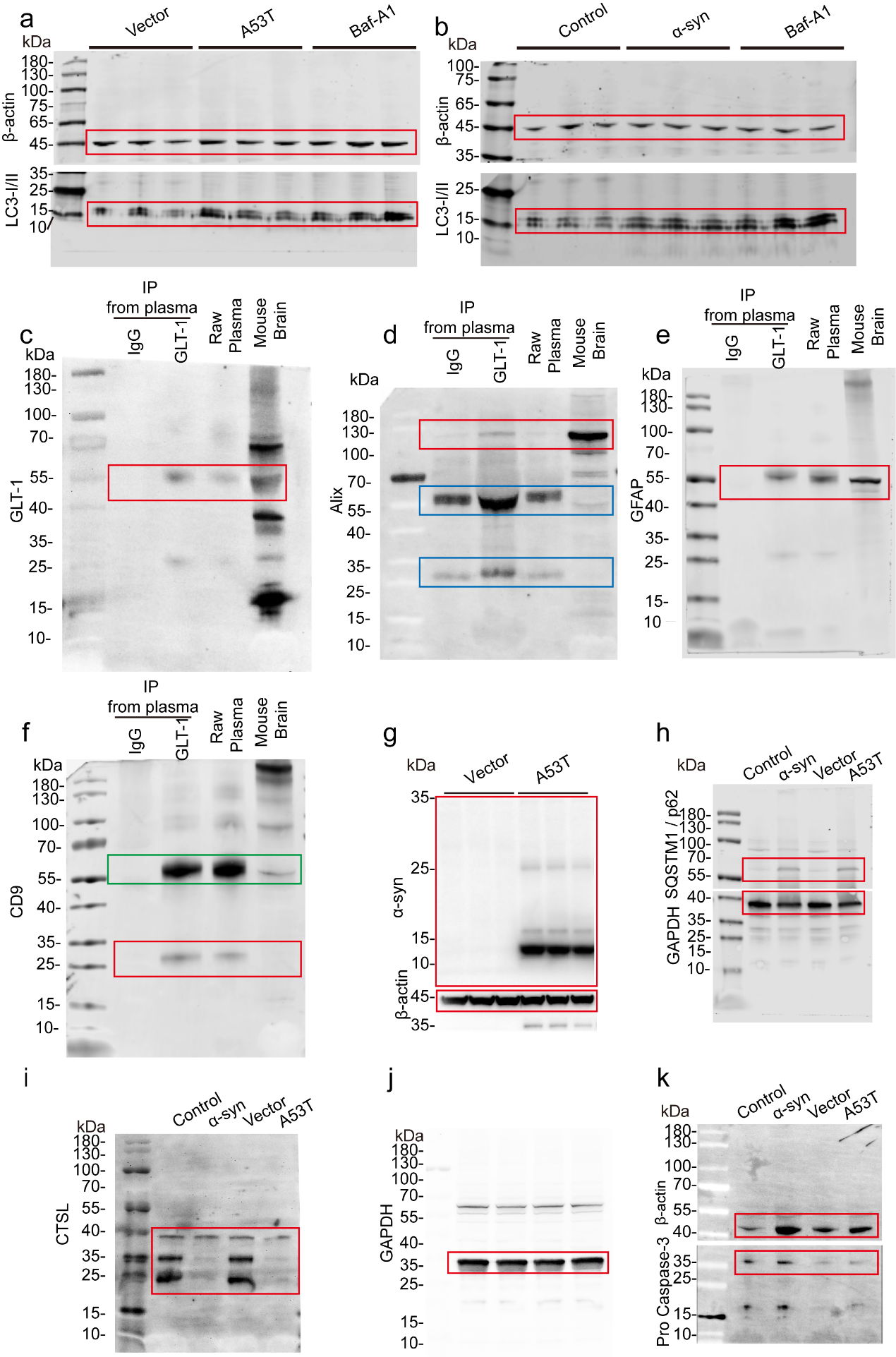


**Figure S4. Full-length Western blots.** (**a**) PVDF membranes were blotted with anti-LC3-I/II and anti-β-actin antibodies. *Red boxes* indicate the cropped images shown in **Fig. 3c**. (**b**) PVDF membranes were blotted with anti-LC3-I/II and anti-β-actin antibodies. *Red boxes* indicate the cropped images shown in **3g**. (**c-f**) PVDF membranes were blotted with anti-GLT-1, anti-Alix, anti-GFAP and anti-CD9 antibodies. *Red boxes* indicate the cropped images shown in **Fig. 4a**. (**g**) PVDF membranes were blotted with anti-α-syn and anti-β-actin antibodies. *Red boxes* indicate the cropped images shown in **Fig. S2a**. (**h**) PVDF membranes were blotted with anti-SQSTM1/p62 and anti-GAPDH antibodies. *Red boxes* indicate the cropped images shown in **Fig. 3i-j**. (**i**) PVDF whole membrane was blotted with the CTSL antibody. *Red boxes* indicate the cropped images shown in **Fig. 2g**. (**j**) PVDF membranes were blotted with the GAPDH antibody. *Red boxes* indicate the cropped images shown in **Fig. 2g**. (**k**) PVDF membranes were blotted with anti-β-actin and anti-Pro Caspase-3 antibodies. *Red boxes* indicate the cropped images shown in **Fig. S3c**.


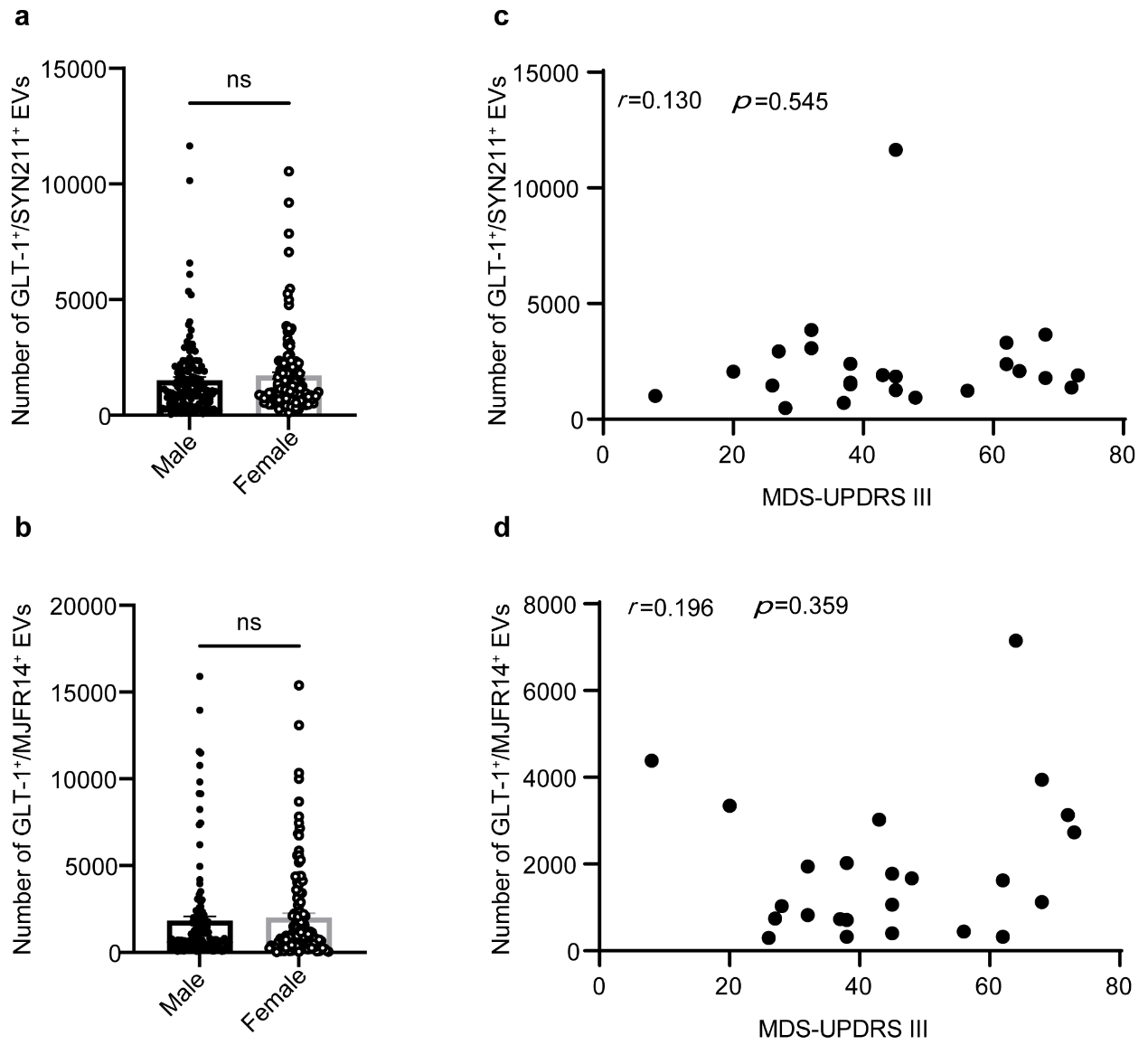


**Figure S5. The correlation of the level of α-syn-carrying astrocytic EVs with gender as well as MDS-UPDRS III.** (**a-b**) The level of AEVs carrying total α-syn (GLT-1^+^/SYN211^+^, **a**) or α-syn aggregate (GLT-1^+^/MJFR14^+^, **b**) has no significant difference in different gender. *N* = 142 in Male group and 114 in Female group. Values are means ± S.E.M., unpaired t-test. ns, not significant. (**c-d**) Spearman's correlation was used to assess the correlation between the level of AEVs carrying total α-syn (GLT-1^+^/SYN211^+^, **c**) or α-syn aggregate (GLT-1^+^/MJFR14^+^, **d**) and MDS-UPDRSIII. *N* = 24.
